# Re-routing MAP kinase signaling for penetration peg formation in predator yeasts

**DOI:** 10.1371/journal.ppat.1012503

**Published:** 2024-08-30

**Authors:** Mareike Rij, Yeseren Kayacan, Beatrice Bernardi, Jürgen Wendland

**Affiliations:** 1 Department of Microbiology and Biochemistry, Hochschule Geisenheim University, Geisenheim, Germany; 2 Research Group of Microbiology (MICR)—Functional Yeast Genomics, Vrije Universiteit Brussel, Brussels, Belgium; 3 Geisenheim Yeast Breeding Center, Hochschule Geisenheim University, Geisenheim, Germany; Universidade de São Paulo Câmpus de Ribeirão Preto: Universidade de Sao Paulo Campus de Ribeirao Preto, BRAZIL

## Abstract

*Saccharomycopsis* yeasts are natural organic sulfur auxotrophs due to lack of genes required for the uptake and assimilation of sulfate/sulfite. Starvation for methionine induces a shift to a predatory, mycoparasitic life strategy that is unique amongst ascomycetous yeasts. Similar to fungal plant pathogens that separated from *Saccharomycopsis* more than 400 million years ago, a specialized infection structure called penetration peg is used for prey cell invasion. Penetration pegs are highly enriched with chitin. Here we demonstrate that an ancient and conserved MAP kinase signaling pathway regulates penetration peg formation and successful predation in the predator yeast *S*. *schoenii*. Deletion of the MAP kinase gene *SsKIL1*, a homolog of the *Saccharomyces cerevisiae ScKSS1/ScFUS3* and the rice blast *Magnaporthe oryzae MoPMK1* genes, as well as deletion of the transcription factor *SsSTE12* generate non-pathogenic mutants that fail to form penetration pegs. Comparative global transcriptome analyses using RNAseq indicate loss of the *Ss*Kil1-*Ss*Ste12-dependent predation response in the mutant strains, while a methionine starvation response is still executed. Within the promoter sequences of genes upregulated during predation we identified a *cis*-regulatory element similar to the *Sc*Ste12 pheromone response element. Our results indicate that, re-routing MAP-kinase signaling by re-wiring Ste12 transcriptional control towards predation specific genes contributed to the parallel evolution of this predacious behaviour in predator yeasts. Consequently, we found that *SsSTE12* is dispensable for mating.

## Author summary

*Saccharomycopsis* yeasts represent a unique yeast genus of predator yeasts that attack and kill fungal prey cells. This necrotrophic mycoparasitism is initiated upon starvation, preferably for organic sulphur/methionine as predator yeasts are methionine auxotrophs. We have characterized morphological and cellular features of predation using time lapse fluorescence microscopy. This indicated prey sensing, chitin enrichment of penetration pegs used in the attack of prey cells and coordination of predation with the cell cycle resulting in cessation of daughter cell growth and a block in mitosis during predation. A predator yeast attack from the onset of penetration peg formation to the disappearance of the nuclear GFP-signal in the *Saccharomyces cerevisiae* prey took ~25 minutes. We identified the MAP kinase *KIL1* and its putative target *STE12* as key virulence genes that govern penetration peg formation. Comparative transcriptomics of mutant strains with the wild type identified specific hunger and predation responses and associated genes. Predation genes were found to harbor a conserved DNA element in their promoters. These findings can aid in deepening our understanding of necrotrophic predator-prey interactions.

## Introduction

A general feature of fungal pathogens is the development of specialized infection structures to force entry into host tissue [1]. These include hyphopodia e.g. in *Verticillium dahliae* or in arbuscular mycorrhizal fungi, infection cushions of *Botrytis cinerea* and *Fusarium graminearum* or appressoria, e.g. in *Magnaporthe oryzae* and *Ustilago maydis* [2–8]. Appressoria are adhesion structures from which penetration pegs emerge to enter the host tissue [9]. Appressoria can be hyaline (*Botrytis*) or melanized as in *Colletotrichum* and *Magnaporthe* [8,10]. Invasion, supported by the synthesis of polymer-degrading enzymes such as cutinases, is achieved by high pressure within melanized appressoria of *M*. *oryzae* to force penetration pegs through the plant cuticle [11,12]. Although the evolutionary origin of appressoria is unknown, molecular details have been obtained by the study of *Magnaporthe* and the basidiomycetous corn smut fungus *Ustilago maydis* [10,12–14]. In both systems MAP kinase signaling plays a central role in appressorium development and subsequent penetration of host tissue. In *Magnaporthe* the *S*. *cerevisiae ScKSS1/ScFUS3* homolog *MoPMK1* regulates appressorium development. Deletion mutants of *MoPMK1* or mutants in which *Mo*Pmk1 can be chemically inactivated do not form appressoria [15,16]. One of the *Mo*Pmk1 targets, the *S*. *cerevisiae ScSTE12* homolog *Mo*Mst12, is also required for virulence since *mst12* mutants form appressoria but fail to generate penetration pegs [17,18]. In *U*. *maydis* the MAP kinases *Umkpp2* and *Umkpp6* are involved in appressorium development; *Umkpp6* mutants can form appressoria, but are defective in penetration [19, 20]. *ScSTE12* homologs have been characterized for their role in virulence in several pathogenic fungi [21–23].

*Saccharomycopsis* deviates from the standard nuclear code by translating the CUG codon as serine instead of leucine; these yeasts are therefore closely related to another CTG clade containing e.g. *Candida albicans*, and to the genus *Ascoidea* [24–26]. Another trait of *Saccharomycopsis* yeasts is their natural methionine auxotrophy due to the loss of several genes for uptake and assimilation of sulfate [27]. Nevertheless, the predator yeast *S*. *schoenii* appears to be rather successful as it attained a global distribution [28]. A striking and unique feature of *Saccharomycopsis* yeasts within the *Saccharomycetales* is their necrotrophic mycoparasitism, i.e. the ability of *Saccharomycopsis* to attack and kill fungal prey cells [28–30]. Interestingly, the killing behavior may not rely on secreted toxins termed ‘killer toxins’ that are a widespread amongst budding yeasts [31,32]. Rather, killing occurs via specialized structures called penetration pegs. The predacious behavior is instigated by starvation, e.g. by removal of methionine from culture media [26,30].

There is a conspicuous resemblance between penetration pegs generated by appressoria of plant pathogenic fungi and those developed by *Saccharomycopsis* despite their large evolutionary distance. This led us to characterize the MAP kinase homolog of *ScFUS3*/*ScKSS1/MoPMK1 in S*. *schoenii*. We demonstrate that this MAP kinase is essential for penetration peg formation and virulence and named the gene *SsKIL1*, mnemonic for killer kinase 1. Similarly, a potential downstream target of *Ss*Kil1, the helix-turn-helix transcription factor of the homeodomain protein family *Ss*Ste12, is required for penetration peg formation. Transcriptome analyses allowed us to separate a methionine starvation induced hunger response from the predation response that is regulated by *SsKIL1* and *SsSTE12*. Three *cis*-regulatory elements were found in differentially expressed genes (DEGs). Our data suggests that within the genus *Saccharomycopsis* parallel evolution resulted in the re-routing of MAP kinase signaling by rewiring Ste12-output to service predation peg formation and the predation response.

## Results

### Penetration pegs can be visualized by fluorescence microscopy

**Fig 1:**
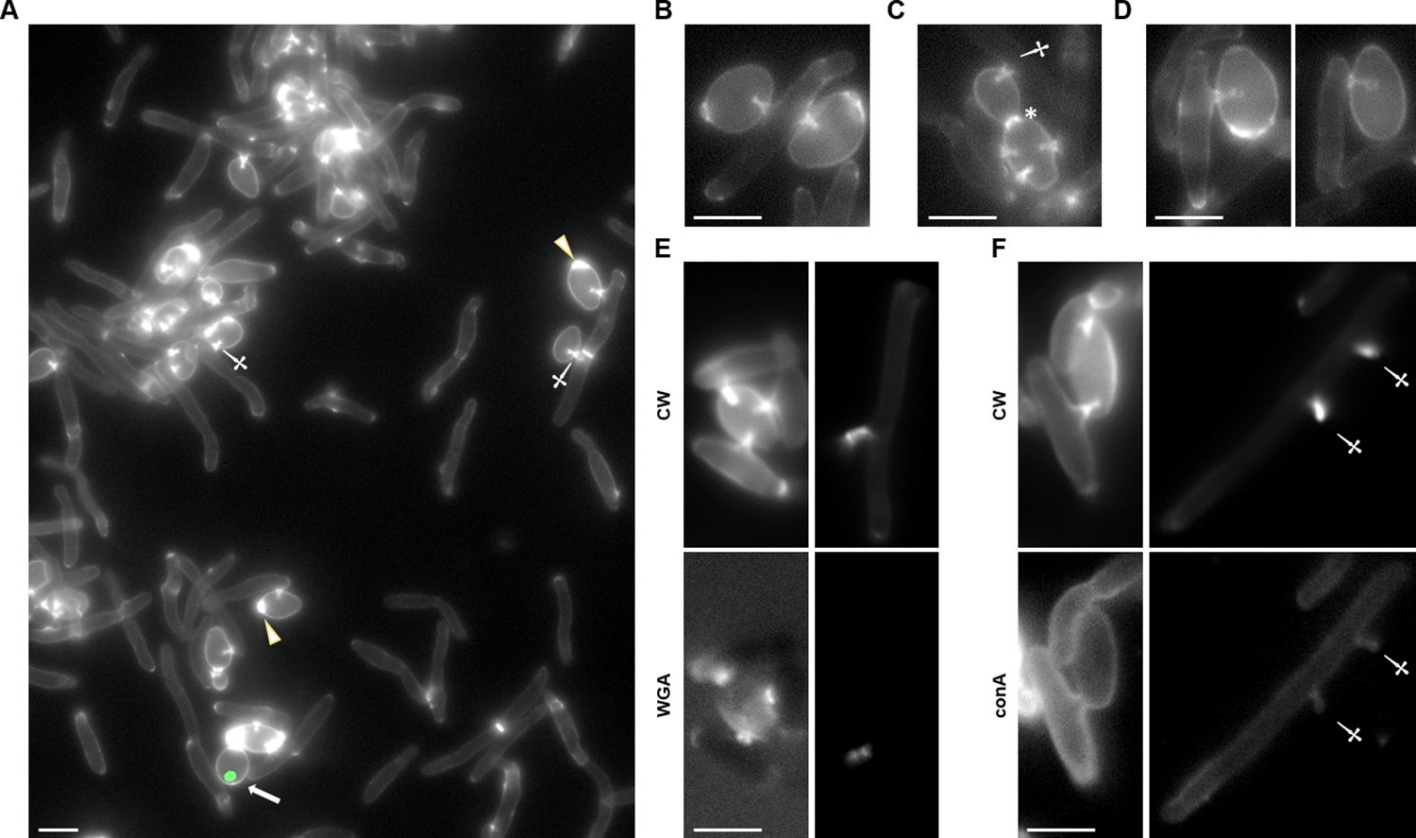
Fluorescence microscopy reveals chitin enrichment of penetration pegs in *S*. *schoenii*. (**A-D**) Live-cell images showing chitin enrichment of calcofluor white (CW)-stained *S*. *schoenii* (elongated) and *S*. *cerevisiae* cells (ovoid). **(A)** Several penetration pegs (two marked by daggers; the left one next to a septum separating mother and daughter *S*. *schoenii* cells) are located inside *S*. *cerevisiae* prey cells. The *S*. *cerevisiae* cells carry a H4-GFP tag but only the one cell at the bottom (marked by an arrow) is showing nuclear fluorescence, while all others do not and are thus dead. *S*. *cerevisiae* bud scars (two positions marked by arrowheads) stain brightly with CW. **(B)** A predator yeast cell (center) with two penetration pegs that attacked two *S*. *cerevisiae* prey cells to the left and right of it is shown. **(C)** A *S*. *cerevisiae* mother cell connected with a daughter cell (the septum between both cells is marked by an asterisk) were attacked simultaneously by different predator cells; one of the four penetration pegs is marked by a dagger. **(D)** Two examples of *S*. *schoenii* predation pegs inside *S*. *cerevisiae* prey cells are shown indicating bulbous tips of the penetration pegs. (**E**) Micrographs showing co-staining of penetration pegs with CW (upper panels, images obtained with DAPI-filterset) and Wheat Germ Agglutinin (WGA CF-488A, lower panels, images acquired with the GFP filterset) of the same cells. (**F**) Images of cells co-stained with CW (upper panels) and concanavalin A (conA CF488A, lower panels). **(E,F)** Panels on the right side show predator-prey cell interactions, while panels on the left depict predator cells without prey cells. (**A-F**), Scale bars, 5 μm.

It has previously been reported that *S*. *schoenii* turns to a predatory lifestyle upon starvation and in the presence of a prey [26], but so far, a more detailed molecular analysis of penetration peg formation has not been reported. *S*. *schoenii* cells are elongated while *S*. *cerevisiae* cells are ovoid, which allows a clear distinction even of unmarked predator and prey cells. Penetration of a prey cell requires removal of its cell wall. To study predator-prey interactions, we used the fluorescent dye calcofluor white (CW), which binds to chitin in the yeast cell wall (Fig 1). In *S*. *cerevisiae* chitin rings at bud scars are brightly stained with CW. Cell wall staining with CW is also brighter in *S*. *cerevisiae* cells compared to *S*. *schoenii* cells. However, predator yeast cells attacking *S*. *cerevisiae* prey cells exhibit strong CW fluorescence of the penetration pegs (Fig 1A). This signal can be used to track predation events and the wild type *S*. *schoenii* strain proved to be a very effective predator yeast against *S*. *cerevisiae*. Histone *H4-GFP*-tagged prey cells lost their nuclear GFP signal after an attack by predator yeast cells and after penetration pegs had entered these cells, indicating their demise (Fig 1A). In Fig 1A there is only one *S*. *cerevisiae* cell that exhibits the nuclear H4-GFP fluorescence, indicating that only this cell is alive while all other prey cells were killed. Predator yeast cells were capable of producing several penetration pegs in their life time and single prey cells could be attacked simultaneously by several predator yeasts (Fig 1A–1C). Prey cells which were not under attack and which were not marked by penetration pegs remained viable. Interestingly, the base of a penetration peg is stained more broadly and the tip of the penetration peg inside the yeast cell sometimes appears to be balloon-shaped with an increased surface area (Fig 1D) [29]. Besides CW, which stains glycosidic bonds, we employed the fluorescent lectin conjugates wheat germ agglutinin (WGA) and concanavalin A (conA) to study penetration peg labelling. Using WGA, which specifically binds to N-acetyl-D-glucosamine found in chitin, only the base of the penetration peg could be labelled but not the region localized within the prey cell (Fig 1E bottom left panel), while CW stains the entire stem of the peg (Fig 1E top left pane). This may be due to the size of the lectin that is far bigger than CW. Therefore, we stained cells with free penetration pegs not surrounded by prey cells. Again, CW stained the entire peg but here also WGA colored the base and tip of the penetration peg (Fig 1E right panels). We then compared CW staining (Fig 1F upper panels) with conA staining (Fig 1F lower panels). The lectin conA, which selectively binds to α-gluco- and α-mannopyranosyl residues, uniformly stained the cell walls of predator and prey cells but did neither label free penetration pegs nor pegs inside prey cells (Fig 1F bottom panels, respectively). This suggests that the penetration pegs are specifically enriched with chitin in their cell wall.

### Analysis of the predation cycle in *Saccharomycopsis schoenii*

**Fig 2:**
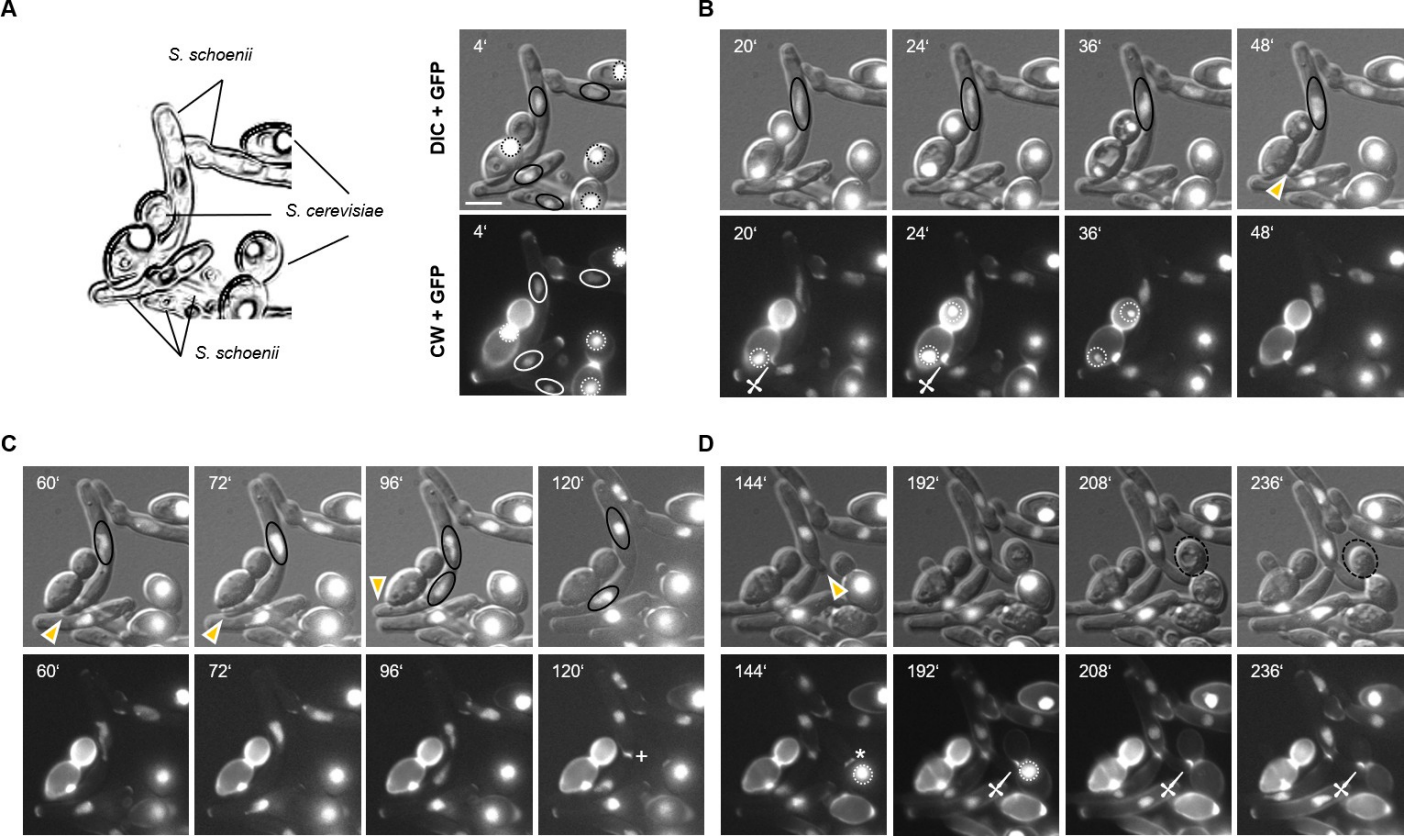
Time-lapse fluorescence microscopy defines distinct stages of the predation cycle in *S*. *schoenii*. **(A)** Schematic drawing of a mixture of elongated *S*. *schoenii* predator and ovoid *S*. *cerevisiae* prey cells at the onset of a time-lapse recording (line drawing on the left based on the DIC + GFP image at 4’). The time series consisted of brightfield images (DIC), GFP-images recording the nuclear *H4-GFP* and CW-images monitoring the appearance of penetration pegs and septa. Upper panels show combined DIC and GFP images, lower panels CW and GFP images. Nuclei of *S*. *schoenii* are encircled with oval lines (black solid lines in the upper images, white solid lines in the lower images). Nuclei of *S*. *cerevisiae* are marked with dashed circles. **(B)** Penetration peg formation is marked by a dagger (20’-24’). *S*. *schoenii* nuclei are marked in the upper panel, while *S*. *cerevisiae* nuclei are marked in the lower panel until their disappearance. The position of the tip of the *S*. *schoenii* daughter cell is marked by an arrowhead. **(C)** Progression of the cell cycle after predation in the predator cell. The growing daughter cell bud tip is marked by arrowheads. *S*. *schoenii* nuclei are encircled in the upper images indicating mitosis at 96’. After mitosis a septum was generated, which is marked by a ‘+`. **(D)** The mother cell entered a new cell cycle marked by bud emergence (arrowhead, 144’). The *S*. *cerevisiae* prey cell is large-budded, its nucleus undivided (marked by a dashed circle) and deposition of chitin at the bud neck is marked by an asterisk. Contact of the predator yeast daughter cell with the prey cell resulted in penetration peg formation (dagger), disappearance of prey cell H4-GFP and shrinking of the prey cell (daughter cell is marked by black dashed circles at 208’ and 236’). Underneath the prey cell killed in **(B)** a predator cell grew **(D)**, which was not involved in the predation of this prey cell and was not the result of further growth of the penetration peg (see also S1 Movie). Scale bar, 5 μm.

Time-lapse microscopy was used to analyze the predation cycle in *S*. *schoenii*. Histone *H4-GFP*-tagged predator and prey cells were mixed on minimal medium agar slides and DIC, CW and GFP images were acquired over time (Fig 2 and S1 Movie). Fig 2 depicts key events during predation for both predator and prey cells. At the onset all cells, predator and prey, show H4-GFP-fluroescence indicating that cells were alive (Fig 2A). A *S*. *schoenii* mother cell with an elongated daughter cell was in contact with a prey cell connected to a large budded daughter cell after mitosis (Fig 2B at 20’). This contact resulted in the formation of a penetration peg (marked by a dagger in Fig 2B at 20’). This initiated predation and paused further growth of the daughter cell and further progression of the cell cycle, i.e. the mother nucleus did not enter mitosis. The CW signal at the penetration peg became more intense (Fig 2B at 24’), while the GFP signal of both mother and daughter prey cells disappeared (Fig 2B between 24’ and 48’). Killing of the prey cells took about 25 minutes from the appearance of a CW signal of the peg until the disappearance of the prey cell nuclear GFP (Fig 2B). Once the prey cells were killed the *S*. *schoenii* daughter cell resumed its polar tip growth at its previous position (Fig 2C at 60’). The mitosis occurred and cell separation was marked by the appearance of a CW-stained septum between the *S*. *schoenii* mother and daughter cells (Fig 2C). Shortly after that the mother cell initiated a new cell cycle (Fig 2D at 144’). The tip of the daughter cell contacted a prey daughter cell and a new penetration peg was formed (Fig 2D at 192’). Killing of the *S*. *cerevisiae* mother and daughter cells occurred within 16 minutes (Fig 2D between 192’ and 208’). After the disappearance of the nuclear GFP-signal prey cells were observed to shrink indicating material transfer into the predator cell (Fig 2D between 208’ and 236’). Thus monitoring predation via time-lapse fluorescence microscopy (n = 29) provided insights into the coordination of both processes. (i) Polarized growth regions of daughter cells were used to generate penetration pegs (24 out of 29) suggesting that polarized growth at the daughter cell can be altered into penetration peg morphogenesis. (ii) Daughter cell growth was halted during predation and resumed at its previous position afterwards indicating maintenance of polarity at the site of bud growth during a predation event (n = 21). (iii) Concomitant with growth arrest of the daughter cell during predation the mother nucleus appeared to be blocked from entry into mitosis suggesting a cell cycle block during predation (n = 9). (iv) This cell cycle arrest clearly indicated that the penetration peg did not receive a nucleus during the predation cycle (n = 29). In fact, we never observed the presence of a nucleus in a penetration peg. This is in contrast to penetration pegs of *Magnaporthe* that further develop into infection hyphae [33]. In *Saccharomycopsis* penetration pegs were only used in a predation cycle and once formed represent a terminal phenotype, i.e. no outgrowth occurred and penetration pegs did not develop into daughter cells. A new predation cycle inevitably required the targeted development of a new penetration peg. (v) Visible hallmarks of a predacious attack were the onset of penetration peg emergence and the diminishing/disappearance of the nuclear GFP-signal in the prey. A predation event could be as fast as 16 minutes. (vi) Killing of prey mother cells also resulted in killing of their connected daughter cells even after mitosis. (vii) Prey cell death was accompanied by shrinking of the prey cell, indicating transfer of nutrients into the predator cell as was observed previously [26].

### MAP kinase signaling regulates penetration peg formation

MAP kinase homologs of the *S*. *cerevisiae FUS3/KSS1* genes have been shown to be essential for fungal development and pathogenicity in a number of filamentous ascomycetes [34,35]. To determine whether a *Saccharomycopsis FUS3/KSS1*-MAP kinase homolog is involved in the predation process we first examined several *Saccharomycopsis* genomes in order to identify MAP kinase genes. This indicated that *Saccharomycopsis* species harbor only a single homolog of the *FUS3/KSS1* MAP kinases similar to *M*. *oryzae*, whereas the more closely related yeast species *Candida albicans* and *Wickerhamomyces anomalus* possess two and in the case of *Ascoidea rubescens* three *FUS3/KSS1* homologs (S1 Fig).

**Fig 3:**
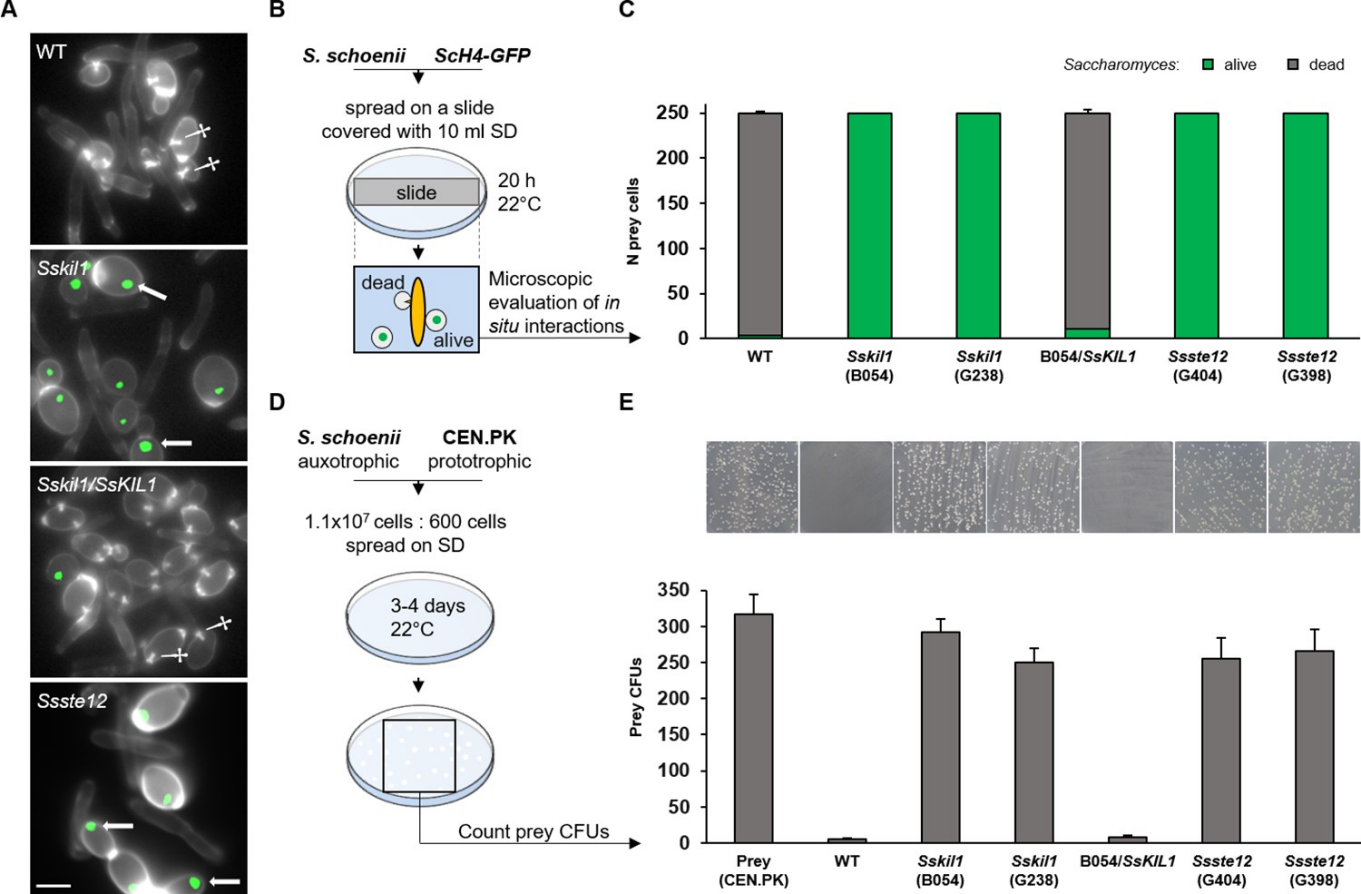
*SsKIL1* and *SsSTE12* are both required for penetration peg formation and predation in *S*. *schoenii*. **(A)** Representative images of predator-prey interactions of the indicated *S*. *schoenii* strains with *S*. *cerevisiae*, *H4-GFP* cells. Cells were stained with CW and CW- and GFP-fluorescence images were acquired and overlaid using ImageJ. Several penetration pegs (absent in *Sskil1* and *Ssste12*) are marked by daggers; several prey cells showing nuclear H4-GFP fluorescence are marked by arrows. WT, wildtype; scale bar, 5 μm. **(B)** Diagram to show the experimental design for *in situ* analysis of predation. Cells of different *S*. *schoenii* strains were mixed with *S*. *cerevisiae H4-GFP* cells and spread on glass slides placed in petri dishes and covered with SD medium. Petri dishes were incubated as indicated. Then glass slides were excised from the matrix, cells were stained with CW and evaluated directly under the microscope. Events scored only included prey cells in direct contact with *S*. *schoenii* cells; dead cells penetrated by a pegs or alive unpenetrated prey cells were quantified. **(C)** Enumeration of predator-prey interactions of the indicated strains prepared as described in **(B)**. WT and *Sskil1/SsKIL1* strains were assayed in triplicate, the *Sskil1* and *Ssste12* mutant strains were assessed by 2 biological replicates and 3 technical replicates per biological replicate, Mean ± s.e.m. **(D)** Experimental design of a plate based predation assay. A lawn of cells of the *S*. *schoenii* tester strain was plated on SD-plates and ~600 *S*. *cerevisiae* prey cells were spread onto this lawn. *S*. *cerevisiae* CFUs were counted within the indicated square. **(E)** Top row shows representative images of petri plates corresponding to the squares shown in **(D)**. *S*. *cerevisiae* CFUs that were formed against the lawn of predator cells enumerated as shown in the graph underneath the corresponding plate images. WT and *Sskil1* (B054) were assayed with 6 biological replicates and 5 technical replicates. *Sskil1* (G238), *Sskil1/SsKIL1* and the *Ssste12* mutant strains were assessed with 3 biological replicates and 5 technical replicates per biological replicate, Mean ± s.e.m.

The complete open reading frame of the single *FUS3/KSS1* homolog in *S*. *schoenii* was deleted via gene replacement cassettes with ~1.5 kb flanking homology regions and two independent mutants were verified by diagnostic PCR (S2 Fig). These mutants did not show any growth defects in standard media. However, when tested in predation assays against *H4-GFP*-tagged *S*. *cerevisiae* prey cells, the *S*. *schoenii* mutants were unable to kill their prey and live prey cells were identified by nuclear GFP-fluorescence (Fig 3A). Mutant *Sskil1* predator cells did not form any highly fluorescent CW-labelled penetration pegs and prey cell invasion could not be observed. Based on this non-virulent phenotype and the decisive role of this gene in the killing of prey cells we named this MAP kinase gene *SsKIL1* for *S*. *schoenii* killer kinase 1. To quantify predation in wild type and *Sskil1* mutants we analyzed cell-to-cell predator-prey interactions *in situ* via fluorescence microscopy. Different from the previous assay, predator and prey cells were incubated on solid medium overnight and were then stained by CW *in situ* and directly observed. Only cells in close contact were analyzed and the dead/alive ratio of prey cells was determined. This indicated that the wild type was a highly efficient predator on *S*. *cerevisiae* prey cells and almost all interactions of starved predator cells with *S*. *cerevisiae* cells ended in the penetration and death of the prey cells. In contrast *Sskil1* strains were completely non-pathogenic. Upon reintroduction of a wild type *SsKIL1* gene in a *Sskil1* strain, predation could be restored to wild type levels (Fig 3B and 3C and S4 Table).

In a reverse assay, a plate-based assay was used to analyze the potential of prey cells to establish colonies against a lawn of predator cells. Predator yeasts, which were themselves unable to form colonies on these minimal medium plates based on their methionine auxotrophy were, however, still able to attack prey cells. Against a lawn of *S*. *schoenii* wild type cells only very few *S*. *cerevisiae* colonies appeared whereas on a lawn of *Sskil1* cells a large number of prey colonies were established indicating lack of predation. In the complemented strain virulence was re-established demonstrating that the defect in predation was caused by *SsKIL1* deletion (Fig 3D and 3E and S4 Table).

### Sensing of the prey and positioning of the penetration peg

**Fig 4:**
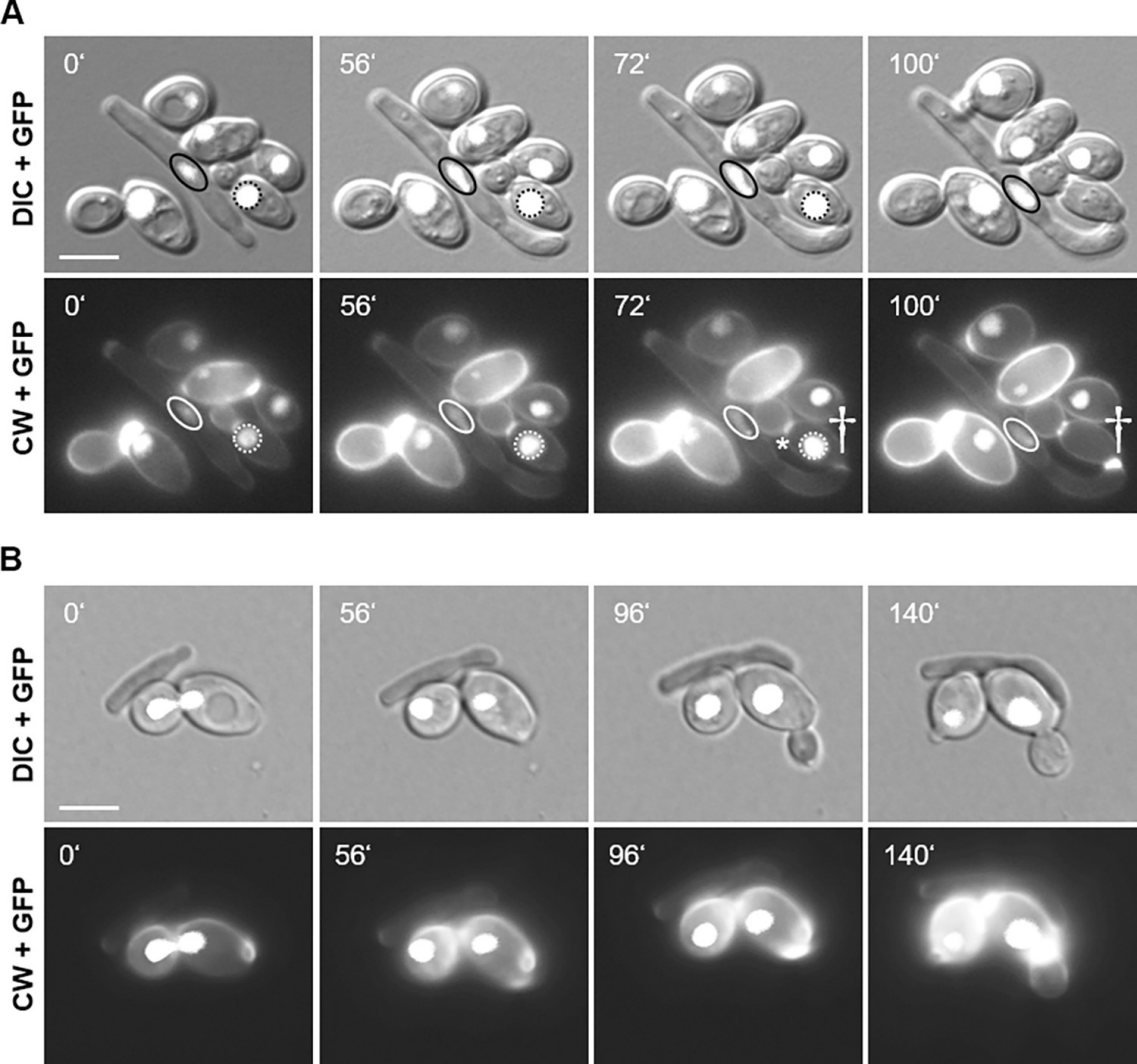
Time-lapse microscopy reveals thigmotropism that preceeds predation in *S*. *schoenii*. **(A)** Selected frames of an *in vivo* time-lapse recording (S3 Movie) showing *S*. *schoenii* (*SsKIL1* wildtype, elongated cell) predation of an *S*. *cerevisiae* prey cell (ovoid cell). Both strains are tagged by histone *H4-GFP* genes to monitor cell viability. The nucleus of *S*. *schoenii* is encircled with oval lines (black solid lines in the upper images, white solid lines in the lower images); the nucleus of the attacked *S*. *cerevisiae* cell is marked with a dashed circle in each frame. Penetration peg formation is marked by a dagger (72’). Note the chitin rich bud neck in *S*. *cerevisiae* (marked by an asterisk at 72’) is present before the penetration peg appears. **(B)** Selected micrographs of a time-lapse recording (S4 Movie) showing the interaction of a *Sskil1* cell (elongated) with a *S*. *cerevisiae* prey cell (ovoid, displaying a nuclear H4-GFP). **(A,B)** At each time point (indicated in minutes, top left in each frame) brightfield (DIC), CW and GFP images were acquired. DIC and GFP images (upper panels) and CW and GFP images (lower panels) were stacked using ImageJ. Strains were incubated on SD medium. Scale bars, 5 μm.

Starvation is a prerequisite of predation, but the factors that regulate positioning of a penetration peg are unknown. Time lapse microscopy indicated that the tip of a growing daughter cell can be utilized to develop a penetration peg (Fig 2 and S1 Movie), however, penetration pegs were also initiated from central regions of a predator yeast cell (S2 Movie). We found directional polarized growth targeted at prey cells in the *S*. *schoenii* wild type (Fig 4A and S3 Movie). Interestingly, such a prey-directed response was also observed in *Sskil1* strains suggesting that this thigmotrophic process is upstream and independent of *SsKIL1* (Fig 4B and S4 Movie). Sensing in *Sskil1* strains, was however, unproductive, did not harm the prey cells and was finally interrupted by cell division.

### *S*. *schoenii Ssste12* mutants are avirulent and do not form penetration pegs

**Fig 5:**
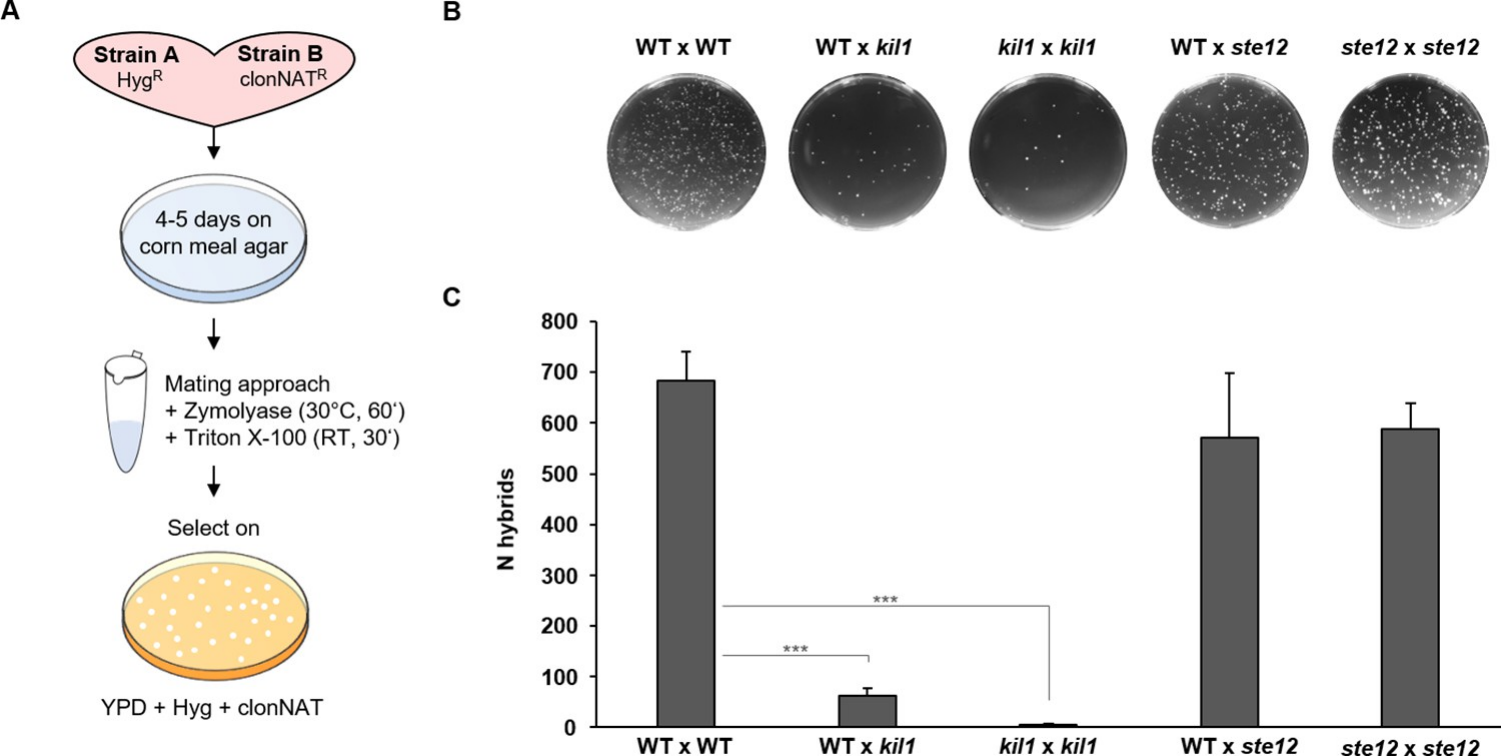
Ss*STE12* is dispensable for mating. **(A)** Experimental design of marker assisted breeding in *S*. *schoenii*. Strains were equipped with either hygromycin (Hyg^R^)or nourseothricin (clonNAT^R^) resistance genes and plated on sporulation media. Spores were enriched by zymolyase/Triton X-100-treatment after wash-off and collection in test tubes and then plated on double selective medium to identify progeny harboring both markers. **(B)** representative images of selection plates of the indicated mating combinations. **(C)** Plot of CFUs (= the number of hybrids, “N hybrids”) identified with the indicated strain combinations. Mating was assessed using 2 biological and three technical replicates for each combination, Mean ± s.e.m.

In *S*. *cerevisiae*, *ScSTE12* is a key downstream transcription factor that is regulated by the *Sc*Fus3/*Sc*Kss1 MAP kinases and controls mating and filamentous growth [36]. The *S*. *schoenii SsSTE12* homolog was identified based on its high conservation to *Sc*Ste12, which is however, limited to the N-terminal region (amino acids 83–256, carrying the Ste-like transcription factor domain pfam02200 with the putative DNA-binding domain). Two *S*. *schoenii Ssste12* deletion mutants were obtained and verified (S3 Fig). *Ssste12* mutants showed no growth defect compared to the wild type. However, when assayed for predation activity *Ssste12* mutant strains were unable to generate penetration pegs and kill prey cells (Fig 3A and S5 Movie). *In situ* analyses of predator-prey cell-to-cell interactions, similar to *Sskil1* strains, showed a complete loss of virulence in *Ssste12* mutants (Fig 3B and 3C). The *S*. *schoenii* wild type is homothallic. However, the strain we used in this study is a poor sporulator. Therefore, we used marker-assisted breeding to compare mating efficiency of *Sskil1* and *Ssste12* strains to the wild type. This indicated that wild type and *Ssste12* strains generate sexual progeny at a similar level. However, *Sskil1* strains, even when mated to a wild type strain, are severely affected in their sexual reproductive capacity (Fig 5 and S5 Table).

### Differential expression analysis identifies loss of the predation response in *Sskil1* and *Ssste12* mutants

**Fig 6:**
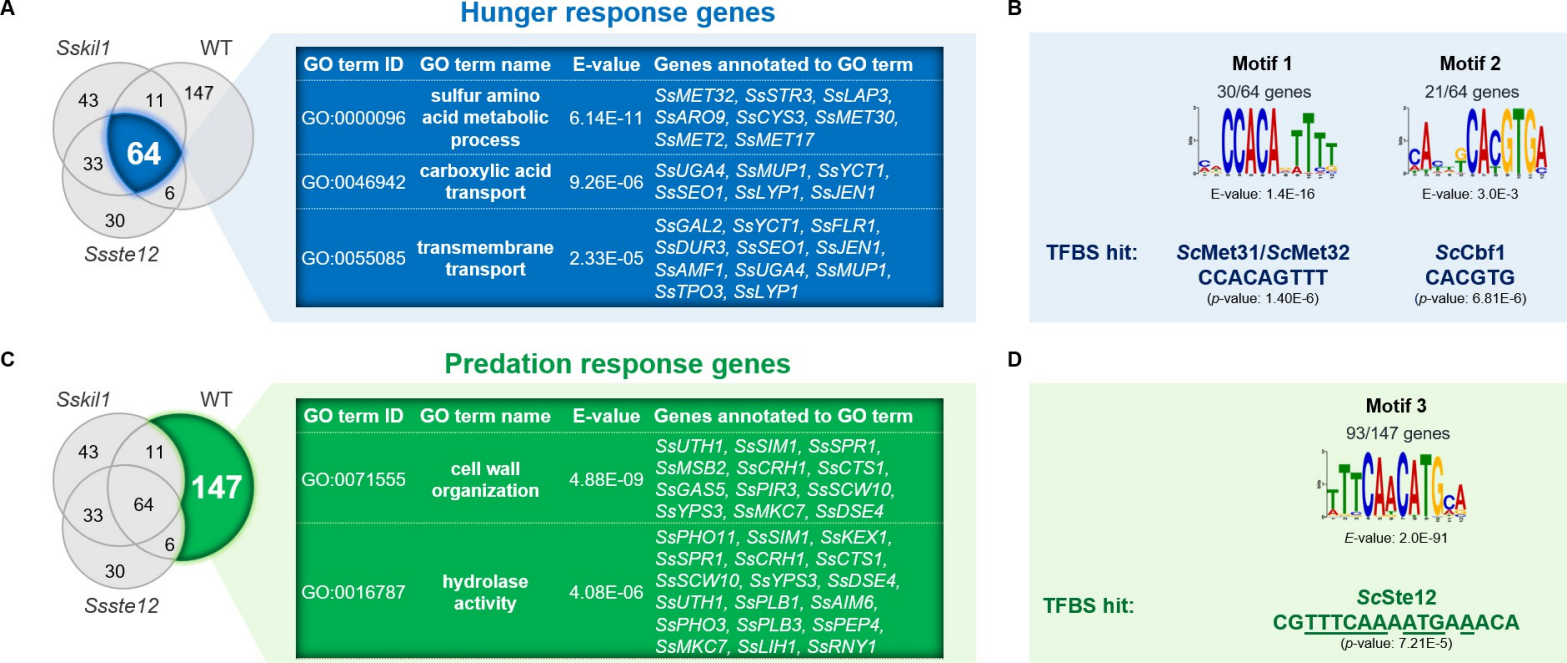
Comparative global transcriptional profiling defines the roles of *SsKIL1* and *SsSTE12* in the predation response of *S*. *schoenii*. **(A)** Venn diagram illustrating overlapping DEGs with at least log_2_ ≥ 2 differential expression under predation conditions between the wild type and the *Sskil1* and *Ssste12* mutant strains. Two distinct gene sets were identified: the hunger response common to all strains **(a)** and the predation response constrained to the wild type **(B)**, each with a set of enriched GO terms. **(C,D)** Consensus DNA-binding motifs, predicted using MEME, for the hunger and predation response gene sets with best matching transcription factor binding site matches (TFBS) to *S*. *cerevisiae* transcriptional regulators.

Previously, proteomics and transcriptomics were used to identify and quantify the *S*. *schoenii* wild type responses during starvation and predation [26]. This defined a starvation and a predation response, which resulted in the identification of enriched GO terms for sulfur metabolic processes and transmembrane transport activities in the starvation response and for GO term categories relating to cell wall organization and hydrolysis in the predation response [26]. The highly similar non-pathogenic phenotypes of *Sskil1* and *Ssste12* strains prompted us to determine the global transcriptional responses of these strains under predation conditions (i.e. on minimal medium lacking methionine) and compare these with each other and to the wild type using a similar transcriptome analysis approach. Differentially expressed genes (DEGs) were called with a *p* value adjusted for a false discovery rate *p*_adj_ < 0.05 and a log_2_-fold change in the expression of genes of log_2_ ≥ 2 to identify upregulated genes (S5 and S6 Tables). This identified 151 DEGs in *Sskil1* and 133 DEGs in *Ssste12* mutant, which we compared to a set of 228 DEGs in the wild type (Fig 6A). A core set of 64 genes was upregulated in all strains. This set encompassed genes involved in sulfur amino acid transmembrane transport with two *SsYCT1* genes (encoding cysteine transporters), *SsMUP1* (a high-affinity methionine permease), the five-member gene family *SsSEO1*encoding putative sulfur compound permeases and genes involved in sulfur metabolism with *SsMET32* (Zinc-finger DNA-binding transcription factor), *SsSTR3* (Peroxisomal cystathionine beta-lyase), *SsLAP3* (Cysteine aminopeptidase), *SsCYS3* (Cystathionine gamma-lyase), *SsMET30* (F-box protein, transcriptional regulator), two *SsMET2* genes (L-homoserine-O-acetyltransferases), *SsMET17* (O-acetyl homoserine-O-acetyl serine sulfhydrylase) as well as genes involved in pyridoxal (vitamin B_6_)-binding, *CYS3*, *MET17* and *STR3*, which require vitamin B_6_ as co-factor in *S*. *cerevisiae* (Fig 6A and S6 Table). *S*. *schoenii* is a pyridoxal-5’-phosphate auxotroph lacking homologs of the *S*. *cerevisiae SNO/SNZ* genes. In line with this observation, we found that *SsTPN1*, encoding a vitamin B_6_-transporter, was upregulated in *Sskil1* and mildly upregulated in wild type and *Ssste12* (S5 Table). As we used minimal medium lacking amino acids for our predation RNAseq approach this hunger response towards organic sulfur deprivation validated our analyses and underlines the specific need of the methionine auxotrophic predator yeasts. Not only genes for the uptake of sulphur compounds were upregulated during the hunger response but also the expression of genes involved in nitrogen uptake was increased, particularly *SsUGA4* (a gamma-aminobutyrate permease), *SsLYP1* (a lysine permease), *SsDUR3* (encoding a plasma membrane transporter for urea and polyamines) and *SsAMF1* (a low affinity NH_4_^+^-transporter) (Fig 6A).

We predicted enriched motifs in the promoter sequences of this commonly upregulated gene set and found two enriched motifs that bear resemblance to the *S*. *cerevisiae* DNA-binding motifs of the Met31/Met32 and Cbf1 transcriptional regulators (Fig 6B and S8 Table). From this data we conclude that the methionine starvation response executed in all strains is independent of either *Ss*Kil1 or *Ss*Ste12.

The largest set of DEGs was associated with genes that were only upregulated during predation in the wild type but neither in *Sskil1* nor in *Ssste12*. This set encompassed 147 genes that describe the predation response (Fig 6C). These DEGs were significantly enriched with genes involved in cell wall organization and biogenesis, particularly with hydrolytic activities such as chitinases (8x *SsCTS1*), glucanases (*SsDSE4*, 2x *SsSCW10* and *SsSPR1*) and aspartic proteases of the yapsin family (11x *SsYPS3*), but also three phospholipase genes (2x *SsPLB1* and *SsPLB3*) (Fig 6C and S6 Table). These GO terms were already previously identified as specifically upregulated in the wild type only in the presence of prey [26]. Within this set of wild-type predation specific DEGs not found in the *Sskil1* and *Ssste12* mutants also other transcriptional regulators, namely Ss*TEC1* and 2x *SsNRG1*, were identified. Analysis of the promoter regions of predation response genes indicated a highly enriched motif (Motif 3) with the consensus sequence ‘TTTCAACATGCA’, which was found in 93/147 predation response genes (Fig 6D and S8 Table). This suggests a central role of *SsSTE12* in mediating the predation response in *S*. *schoenii*. Reporter gene analyses with two predation genes (Ssc08w0210-*YPS3* and Ssc02c0119-*CTS1*) indicted that their expression under predation conditions is dependent on Ste12 (S4 Fig).

The predator-prey interaction period we chose in our experiment was 1.5 h. Under this condition we found only a very small set of 17 downregulated DEGs in the wild type. Nine of these genes were also found to be downregulated in *Sskil1* and *Ssste12*. These genes are involved in metal ion transport and include *SsFRE7*, a putative ferric reductase and *SsCTR1*, a high-affinity copper transporter. In contrast, in *Sskil1* 120 DEGs and in *Ssste12* 111 DEGs were found to be downregulated with an overlap of 44 shared DEGs. In both mutant strains the dominant sets of DEGs were involved in ribosome biogenesis and rRNA processing (S9 Table). Interestingly, we did not observe this stress response in the wild type indicative of wild type growth after predation.

## Discussion

In this study we have identified the MAP kinase gene *SsKIL1* as the key regulator of the predation response in the mycoparasitic yeast *S*. *schoenii*. Using phenotypic characterization and global transcript profiling, we determined a large overlap in *Sskil1* mutant phenotypes with its conserved and putative downstream target *SsSTE12*. Both factors orchestrate the activation of genes required for penetration peg formation and the expression of cell wall degrading enzymes supporting the attack of prey cells. Deletion mutants in *SsKIL1* and *SsSTE12* are, therefore, non-pathogenic. In *S*. *cerevisiae* the mating and starvation/filamentation pathways are controlled by the MAP Kinase genes *ScFUS3* and *ScKSS1*, respectively, and a central downstream target of these MAP kinases is the homeodomain transcription factor *Sc*Ste12 [37]. Furthermore, this MAPK cascade is highly conserved in the fungal kingdom and has been shown to regulate pathogenicity in a wide variety of fungal pathogens [21].

The *Magnaporthe Sc*Ste12 homolog *Mo*Mst12 is a direct target of *Mo*Pmk1, which regulates the formation of melanized appressoria through activation of the transcription factor *Mo*Hox7 [18,38]. Additionally, *STE12* and *STE12*-like genes (with additional carboxy-terminal C_2_H_2_ zinc fingers) have been shown to regulate sexual development, e.g. in *Aspergillus* and *Neurospora*, and pathogenicity in a wide variety of fungal systems including, for example, *Botrytis cinerea*, *Candida albicans*, *Cryptococcus neoformans*, *Colletotrichum* and *Fusarium* [21–23].

*Saccharomycopsis* species, in contrast to other members of the *Saccharomycotina*, possess only a single MAP kinase homolog of *ScFUS3* and *ScKSS1* (S1 Fig). Our study indicated that *S*. *schoenii SsSTE12* is dispensable for mating and thus dedicated to the predation process. Motif 3 ‘TTTCAACATGCA’ that was present in a large number of predation response gene promoters bears similarity to a homodimeric *S*. *cerevisiae Sc*Ste12-binding site [39]. This suggests direct activation of a large part of the predation response genes via *SsSTE12*. Our study revealed additional transcriptional regulators, *SsTEC1* and *SsNRG1*, that may act as downstream targets of *Ss*Kil1/*Ss*Ste12 to orchestrate penetration peg formation. In *S*. *cerevisiae* the activation of filamentation genes requires both *ScSTE12* and *ScTEC1* [40]. *S*. *schoenii* has a single homolog of *SsTEC1*. *SsTEC1* contains one copy of Motif 3 and is strongly upregulated in wild type during predation (log_2_ = 3.14), but only mildly upregulated in *Sskil1* and *Ssste12* (log_2_ between 1.15 and 1.46). This suggests that a subset of predation genes may depend on both *Ss*Ste12 and *Ss*Tec1 for activation in *S*. *schoenii*. Another transcriptional regulator that was strongly upregulated in *S*. *schoenii* wild type was *SsNRG1*. There are five *SsNRG1* paralogs in *S*. *schoenii*, three of these were upregulated in the wild type, but none in *Sskil1* (S6 Table). In *S*. *cerevisiae ScNRG1* is a transcriptional repressor e.g. of filamentous growth [41].

The predation cycle in *S*. *schoenii* was more clearly defined by this study. Predation in *Saccharomycopsis* requires starvation as unstarved cells do not engage in predation even in the presence of prey [26,42]. Starvation in the methionine auxotroph predator yeasts can easily be invoked by methionine deprivation as demonstrated. The starvation response we observed in our study centered on the expression of sulfur uptake and metabolism genes. In these genes conserved motifs were found that bear similarity to *Sc*Met31/*Sc*Met32 and *Sc*Cbf1 binding sites, which are *cis-*acting regulatory elements of genes involved in sulfate assimilation and methionine biosynthesis in *S*. *cerevisiae* [43]. The methionine starvation response was independent of *SsKIL1/SsSTE12*. The *Sskil1* and *Ssste12* mutants showed an additional much stronger response to starvation than the wild type in our assay found in the downregulation of a large set of ribosomal genes similar to the stress response observed in *S*. *cerevisiae*, which under nutrient stress conditions also results in the repression of ribosomal protein genes [44]. This indicates that the wild type rapidly—within the 1.5 h timeframe of our experiment- benefits from predation and quickly redeems the cost of penetration peg formation. Investigations on how hunger primes predation may provide insight into the upstream regulation of MAP kinase signaling in *S*. *schoenii*. In *Magnaporthe* the G-protein coupled receptor *Mo*Pth11 is required for appressorium differentiation induced by hydrophobic signals and acts upstream of the cAMP signaling pathway [45]. In predator yeasts sensing and recognition of a prey cell occur and tropism towards a prey cell is apparently independent of *SsKIL1*. Components acting upstream of *SsKIL1* could be involved in nutrient chemotropism, i.e. the sensing of carbon or nitrogen sources linked to a prey cell [46,47]. In *Candida albicans* thigmotropism was shown to be regulated by the Ras-like GTPase *CaRSR1* [48]. Investigating nutrient transceptors like *SsGPR1* and the Ras-like GTPase *SsRSR1* in *Saccharomycopsis* may be helpful to elucidate predator-prey interactions.

Penetration pegs formed by predator yeasts resemble penetration pegs generated by appressoria of plant pathogenic fungi, two groups of fungi that separated more than 400 million years ago [49]. This is a remarkable example of parallel evolution and shows the inherent plasticity of the Fus3/Kss1-MAP kinase cascade in fungi. However, predator yeast penetration pegs differ from e.g. *Magnaporthe* penetration pegs in that they are enriched with chitin, which may act as a shield against the cell wall lytic enzymes used in the attack of the prey. Furthermore, penetration pegs are a dead end: they are a one-off investment in mycoparasitism. Predator yeast penetration pegs do not receive a nucleus and thus do not develop into daughter cells or into nucleated infection hyphae as in *Magnaporthe* [50]. On the contrary, during predation we observed a halt in daughter cell growth and a block of the cell cycle. Both activities were resumed after killing of the prey indicating a coordinated regulation of cell cycle progression and predation. In the corn smut fungus *Ustilago maydis* a cell cycle arrest in G2 has been described that is required for appressorium formation [13]. *Saccharomycopsis* species are successful in attacking a diverse set of yeasts and filamentous fungi. A broad host range has also been observed in other necrotrophic fungi, for example in *Trichoderma* [42,51,52].

In summary, we have provided a detailed characterization of the predation cycle in mycoparasitic predator yeasts and demonstrated that *S*. *schoenii* predacious interactions are regulated by the MAP kinase *Ss*Kil1 and its putative target *Ss*Ste12, which act as global regulators of penetration peg formation required for invasion of prey cells. The *SsKIL1/SsSTE12* signaling pathway controls predation response genes, which includes large families of cell wall lytic hydrolases, particularly chitinases, glucanases and proteases, but also additional transcription factors. Predator yeasts, therefore, represent a unique and simple unicellular system to study host-pathogen interactions and pathogenicity mechanisms that have evolved in parallel to those of distant filamentous plant-pathogenic ascomycetes.

## Materials and methods

### Fungal strains, growth conditions and culture conditions

All strains used and generated in this study are stored in the laboratory collections (Dept. of Microbiology and Biochemistry, Geisenheim University). Strains and their genotypes are listed in S1 Table. Strains were propagated in YPD (1% yeast extract, 2% casein peptone, 2% glucose;) at 30°C. Antibiotic resistance against nourseothricin (clonNAT), geneticin (G418) and hygromycin was used to select transformants, deletion mutants and hybrids. Selective media contained 100 μg/ml clonNAT, 200 μg/ml G418 and/or hygromycin (Genaxxon bioscience GmbH, Ulm, Germany). Predation was induced on minimal media plates using either SD (1.7 g/L yeast nitrogen base (YNB) without amino acids, 5 g/l NH_4_SO_4_, 20 g/L glucose) or CSM (SD + 0.69 g/L CSM complete synthetic medium; Formedium Ltd, Norfolk, UK). Solid media contained 2% agar. *Escherichia coli* DH5α was used for plasmid propagation in 2xYT (1.6% bacto peptone, 1% yeast extract, 0.5% NaCl) with 100 μg/ml ampicillin at 37°C. *Saccharomyces cerevisiae* strain BY4741 was used for *in vivo* cloning.

### Generation of targeted deletion mutants

Gene sequences were obtained from the *S*. *schoenii* draft genome sequence [26]. All primers were obtained from Sigma-Aldrich, Taufkirchen, Germany) and sequences of the primers used are provided in S2 Table. Plasmids used and generated in this study are listed in S3 Table. Gene replacement cassettes were either generated by *in vivo* recombination in *S*. *cerevisiae* or were custom synthesized. To construct the *SsKIl1* complete ORF deletion cassette homology regions (HRs) of ~1.5 kb upstream and downstream of the *SsKIL1*-ORF were PCR-amplified and cloned into pGEM (Promega, Madison, USA) using primers #44 and #45 for the upstream and #46 and #47 for the downstream HR. Adapter sequences and *Sma*I restriction sites were added to the *SsKIL1* HRs by PCR using primers #48 and #49 for the 5’-HR and #50 and #51 for the 3’-HR. These PCR-fragments along with *SAK1* marker gene [53] (amplified from pGEM-*SAK1* with primers #96 and #97) and the *SmaI*-linearised pRS415 vector were co-transformed into *S*. *cerevisiae* using the LiAc/single-stranded carrier DNA/PEG method with DMSO [54,55]. Correct assembly of the plasmid was verified by diagnostic PCR with primers #48 and #49 for 5’-HR, #50 and #51 for 3’-HR and #18 and #7272 for the internal region of *SAK1*. The *SsSTE12* complete ORF deletion cassette was obtained from BioCat (Heidelberg, Germany). All plasmids were propagated in *E*. *coli* strain DH5α. Plasmid DNA was extracted using the PureYield Plasmid Midiprep System (Promega, Walldorf, Germany). *SsKIl1* disruption cassette was excised via *Sma*I and *SsSTE12* disruption cassette was amplified as full-length fragment from plasmid DNA using primers #1022 and #1023. Targeted gene deletion in predator wild type strain was achieved as described [56]. Deletion mutants were verified via diagnostic PCR using G1-G2, G3-G4 and I1-I2 primer pairs #80-#7269, #7270-#81, #82-#83, respectively, for *Sskil1* and primer pairs #1020-#1026, #7270-#1021, #1018-#1019 for *Ssste12* (S2 and S3 Tables).

### Generation of strains for complementation, GFP-expression, marker assisted breeding and promoter analysis

For complementation of the *Sskil1* defect a cassette was constructed by *in vivo* cloning in *S*. *cerevisiae*. To this end the pRS415-*YES2* plasmid was linearized with *Sma*I and co-transformed into *S*. *cerevisiae* with a PCR product generated from *S*. *schoenii* genomic DNA with primers #445 and #447 containing the *SsKIL1* gene. Correct assembly was verified by PCR. The obtained plasmid was linearized with *Eco*RV and transformed into *Sskil1* strain B054. Plasmid E196 carrying a synthetic *S*. *schoenii* histone *H4-GFP* fusion gene, obtained from Genscript (Leiden, Netherlands), was linearized by *Sac*I digestion and transformed into *S*. *schoenii*. For marker-assisted breeding strains to be mated were equipped with either hygromycin or nourseothricin resistance marker genes to allow for double selection of progeny. To this end PCR-fragments of the marker genes were amplified from *YES2* (E068) and *YES3* (E070) plasmids using primer pairs #285-#287 and #1003-#1004, respectively, and transformed into the selected *S*. *schoenii* strains.

For conditional expression analyses, the *S*. *schoenii* promoters *SsYPS3*p (Ssc08w0210) and *SsCTS1*p (Ssc02c0119) were fused to *lacZ* reporter gene by *in vivo* cloning in *S*. *cerevisiae*. For this purpose, plasmid E074 (pRS417-*SsMET17*p-*lacZ*-*SAK1*, [53]) was cleaved with restriction enzymes *Sma*I, *Xho*I, *Pst*I and *Sac*I. The hygromycin resistance marker was amplified using primer pairs #1272-#107 and #251-#1273, and the promoters were amplified from *S*. *schoenii* genomic DNA with primers #1251 and #1252 for *SsYPS3*p and primers #1256 and #1257 for *SsCTS1*p. Correct plasmid assembly was verified by selecting *S*. *cerevisiae* transformants with hygromycin indicating successful replacement of the resistance marker and by PCR with primer pairs #1253-#223 and #1258-#223 and to test for *SsYPS3*p- and *SsCTS1*p-*lacZ* fusion, respectively. This resulted in plasmids E324 (pRS417-*SsCTS1*p-*lacZ*-*YES2*) and E326 (pRS416-*SsYPS3*p-*lacZ*-*YES2*), which were each linearized with *SacI* and transformed into the selected *S*. *schoenii* strains.

### Predation assays

An array of assays was used to analyze predacious interactions of *S*. *schoenii* strains with its prey *S*. *cerevisiae*, *ScH4-GFP*. Standard predation assays were performed by mixing equal amounts of predator and prey cells spread on SD-plates. Interactions were allowed to proceed for up to 20 h at 22°C. For subsequent microscopic observations, cells were washed off the plates after 4 h and stained with CW prior to imaging. For direct *in situ* analyses of predator-prey interactions, objective slides covered with 10 ml SD medium were used onto which predator and prey cells were spotted, and incubated for 20 h at 22°C. Cells were stained with CW and for each strain 250 predator-prey-interactions were evaluated microscopically to quantify the ratio of prey cells attached to predator cells that were alive (i.e. predator cells formed no penetration pegs and prey cells were alive according to their GFP signal) or penetrated and dead (predator cells formed pegs and prey cells lost their GFP signal). Samples were prepared in triplicates. In plate-based assays, ~ 600 cells of the prototrophic prey strain *S*. *cerevisiae CEN*.*PK* were trialled against a lawn of predator cells (~1.0x10^7^ cells) on SD medium for 3–4 days at 22°C. Predator yeasts are methionine auxotrophs and thus unable to proliferate on these plates, while the prototrophic prey strain may form colonies if no predation has occurred. Plates were imaged using a Canon EOS 77D camera equipped with an EF-S 18–135 mm objective. Prey CFUs were determined by counting. The assays were performed for three to six biological replicates with five technical replicates and results are provided as the mean ± s.e.m.

### Mating assay

In these assays, 2.5 x 10^7^ cells of each strain were mixed, plated on corn meal agar (Merck KGaA, Darmstadt, Germany) and incubated for 4–5 days at 22°C. Then cells were washed off the plates, treated with 1.6 mg/ml zymolyase (Genaxxon bioscience GmbH, Ulm, Germany) for 1 h at 30°C and then with 0.04 mg/ml Triton X-100 for 30 min at RT to eliminate vegetative cells. Samples were washed three times with H_2_O, resuspended in 750 μl H_2_O of which 1/10 volume was plated on double selective YPD medium containing hygromycin and nourseothricin to select for hybrid progeny expressing both marker genes. Two biological replicas were assayed with three technical replicates for each strain combination.

### Microscopic analyses

Microscopic images were acquired either by an Axiovert 200M (Zeiss, Jena, Germany) microscope equipped with an AxioCam MRm camera run by the Axiovision 4 software package (Zeiss, Jena, Germany) or using an Axio Imager with a pco.edge 4.2bi camera controlled by the VisiView 6 software (Visitron Systems GmbH, Puchheim, Germany). For *in vivo* time-lapse microscopy, predator strains and *S*. *cerevisiae H4-GFP* were mixed on deep well slides containing solidified CSM medium supplemented with 5 μg/ml CW. Image acquisition was automated with DIC images taken every 2 min and GFP and CW fluorescence images every 4 min. Appropriate excitation and emission Chroma ET filter sets were used with LED illumination. Image processing was done using ImageJ 1.53c. Images of similar treated cells or all single plane files of a time-lapse series were stacked channel-wise and processed equally. We used the ImageJ plugins ‘NMS_fixTranslation_ver1.ijm’ for drift correction and ‘SPIM_DrawArrowInMovie_-14.0.0.jar‘ to add arrows to a movie.

### Staining procedures

Yeast cell walls were stained with the fluorescent dyes calcofluor white (CW) or the fluorescent lectin conjugates Wheat Germ agglutinin (CF-488A Wheat germ agglutinin, WGA) or concanavalin A (CF-488A, conA). Dyes were purchased from VWR International GmbH (Darmstadt, Germany). Cells were treated with 0.01 mg/ml CW or co-stained with CW and up to 0.15 mg/ml WGA or conA. Predator wild type cells form penetration pegs even in the absence of prey when cultivated on corn meal agar plates. These cells were washed off the plates, resuspended and co-stained with CW and 0.1 mg/ml WGA or conA.

### MAPK Alignment

Genomes of the species analysed were queried against *S*. *cerevisiae* the Kss1 protein sequence using either blastp or tblastn (https://blast.ncbi.nlm.nih.gov/Blast.cgi) (S1 Fig). Hits were retrieved and analyzed using the scan prosite tool (https://prosite.expasy.org/). Proteins that carried a MAP kinase signature (PS01351) were reciprocally blasted against *S*. *cerevisiae* using blastp at the *Saccharomyces* Genome Database (SGD; https://www.yeastgenome.org/). The Hog1 MAP kinase sequences were used to generate a protein tree using MegAlign (DNASTAR, Madison, WI, USA).

### Transcript profiling via RNAseq analysis

For transcriptomics, 2.27x10^7^ cells of *S*. *schoenii* wild type (CBS 7425), *Sskil1* (B054 and G238) and *Ssste12* (G404 and G398) were cultured either alone on YPD (starvation/predation suppressing medium) or co-cultured with the same amount of *S*. *cerevisiae* BY4741 prey cells on SD (starvation/predation inducing medium) for 1.5 h. Cells were collected from these plates and frozen in liquid nitrogen. RNA extraction, quality control, library preparation (PolyA selection with ERCC spike-in), sequencing at 30M paired-end (2x150 bp) reads/sample using Illumina NovaSeq and standard data analysis were performed at Genewiz (Azenta Life Sciences, Leipzig, Germany). Genes with *p*_adj_ < 0.05 and a 4-fold change in expression level on hunger/predation inducing conditions (SD) compared to suppressing conditions (YPD), i.e. upregulation (log_2_ ≥ 2) and downregulation (log_2_ ≤ -2), were defined as differentially expressed genes (DEGs). The overlapping sets of DEGs of biological replicates of *Sskil1* B054 and G238 as well as *Ssste12* G398 and G404, respectively, were compared to DEGs of the wild type.

### Gene set analyses

Subsets of unique and co-regulated DEGs displayed in Fig 6 were subjected to enrichment analyses using the generic gene ontology (GO) term finder (https://go.princeton.edu/cgi-bin/GOTermFinder) with default settings. GO terms associated with biological processes, molecular function and cellular components with a *p*-value < 1.0E-02 were considered to be significantly enriched. Redundancy among GO terms were reduced using REVIGO v 1.8.1 in default settings and the *S*. *cerevisiae* S288C database (http://revigo.irb.hr/).

### Motif enrichment analysis

Motif discovery in promoter sequences (defined as up to -1 kb of the start codon) of upregulated DEGs with log_2_ ≥ 2 was carried out using MEME (https://meme-suite.org/meme/index.html). Motif searches were limited for sequences ranging between 6 to 24 bases with multiple but non-overlapping occurrences. Letter probability matrices of significantly enriched motifs were subjected to Tomtom v 5.5.5 using YEASTRACT database (https://meme-suite.org/meme/tools/tomtom). Hits of query motifs to a transcription factor binding site (TFBS) identified in *S*. *cerevisiae* with a *p*-value < 1.0E-04 were reported.

### Promoter analysis

The regulation of the *S*. *schoenii* promoters Ssc08w0210-*YPS3*p and Ssc02c0119-*CTS1*p under predation conditions was assayed using the *lacZ* reporter gene. *LacZ* expression was examined in predator strains G538 (WT, *SsCTS1*p-*lacZ*), G550 and G551 (*Ssste12*, *SsCTS1*p-*lacZ*), G556, G557 and G558 (WT, *SsYPS3*p-*lacZ*) and G560, G561 and G562 (*Ssste12*, *SsYPS3*p-*lacZ*) when cultivated alone on YPD (predation suppressive) or with *S*. *cerevisiae* BY4741 prey cells on SD (predation inducing) similarly as done for the RNAseq analyses. For comparison G218 (WT, *SsTEF1*p-*lacZ*) was used as strain with constitutive expression. The β-galactosidase activity was then determined by a liquid phase ONPG (ο-nitrophenyl galactopyranoside) assay as described previously [57].

### Statistics and reproducibility

Datasets of mating experiment were subjected to Shapiro-Wilk normality test which yielded p-values > 0.05 indicating normal distribution of data. A one-sided Welch’s unpaired t-test was applied and significant results were considered at p-values < 0.05.

Transcriptomes of *S*. schoenii wild type and its derivative strains analyzed in this study have been submitted to NCBI BioProject database under the accession ID “PRJNA1091439”.

All experiments were conducted with at least two biological replicates and technical replicates of an appropriate sample size, estimated based on what is established in the field. The sample sizes, number of biological and technical replicates, and the statistical tests used in each experiment are specified in the figure legends.

## Supporting information

S1 Fig MAP kinase conservation in yeast. Protein tree based on Hog1 protein sequences was generated using MegAlign (DNASTAR, Madison, WI, USA) to show conservation of MAP kinase genes across diverse yeast species of *Pachysolen tannophilus* NRRL Y-2460 (bio project PRJNA69545), *Wickerhamomyces anomalus* NRRL Y-366-8, (PRJNA60493), *Komagataella pastoris* (PRJNA942376), Saccharomyces cerevisiae (https://www.yeastgenome.org/), *Candida albicans*, (http://www.candidagenome.org/), *Debaryomyces hansenii* CBS767 (PRJNA12410), *Hanseniaspora uvarum* QTX-C10 (PRJNA954297), *Ascoidea rubescens* DSM 1968 (PRJNA207865), *Saccharomycopsis schoenii* CBS 7425 (PRJNA251344), *Saccharomycopsis fermentans* CBS 7830 (PRJNA251344), *Saccharomycopsis vini* CBS 4110 (PRJNA977123), *Saccharomycopsis crataegensis* CBS 6448 (PRJNA977123), *Saccharomycopsis olivae* CBS 12701 (PRJNA736342), *Saccharomycopsis fodiens* CBS 8332 (PRJNA251344), *Saccharomycopsis synnaedendra* CBS 7763 (PRJNA977123). MAP kinases were identified using blast searches of genome sequences at NCBI (https://blast.ncbi.nlm.nih.gov/Blast.cgi). Map kinase signatures were predicted using the scan prosite tool (https://prosite.expasy.org/). Proteins that carried a MAP kinase signature (PS01351) were reciprocally blasted against *S*. *cerevisiae* using blastp at the *Saccharomyces* Genome Database (SGD; https://www.yeastgenome.org/). Smk1 protein orthologs were identified using *Sc*Smk1 as query in blast searches.
(TIF)

S2 Fig Deletion of the *Saccharomycopsis schoenii KIL1* gene. (**A**) Schematic representation of the *SsKIL1* locus with adjacent gens, the disruption cassette containing *SAK1*, a dominant selectable marker gene providing resistance against [53] and the *SsKIL1*-locus after deletion of the *SsKIL1*-ORF and integration of the cassette. The 5’- and 3’-homology regions are marked as well as locus- and marker-specific primers used diagnostic PCRs to verify correct integration with the indicated expected sizes. (**B**) Gel image with diagnostic PCRs of the *Sskil1* mutants and the complemented strain compared to wild type. Correct transformants showed G1-G2 and G3-G4 bands. *Sskil1* mutants lacked the I1-I2 *SsKIL1*-internal band, which is present in the wild type and the complemented strain.
(TIF)

S3 Fig Deletion of the *Saccharomycopsis schoenii STE12* gene. (**A**) Schematic representation of the *SsSTE12* locus with adjacent gens, the disruption cassette containing *kanXS*, a synthetic dominant selectable marker gene consisting of the *SsPGK1* promoter and the kanamycin resistance ORF derived from *YES1* [53] and the *SsKIL1*-locus after deletion of the *SsSTE12*-ORF and integration of the cassette. The 5’- and 3’-homology regions are marked as well as locus- and marker-specific primers used diagnostic PCRs to verify correct integration with the indicated expected sizes. (**B**) Gel image with diagnostic PCRs of the *Ssste12* mutants and the complemented strain compared to wild type. Correct transformants showed G1-G2 and G3-G4 bands and lacked an I1-I2 *SsSTE12*-internal band, which is present in the wild type.
(TIF)

S4 Fig Promoter activity of predation response genes. Promoters of the predation response genes Ssc08w0210-*YPS3*p and Ssc02c0119-*CTS1*p were tested for conditional regulation under predation conditions using a liquid-phase β-galactosidase assay. *Saccharomycopsis* strains were trialed on YPD without prey cells (repressive conditions) and on SD with prey cells (inductive conditions) as described in *Materials and methods*. The *lacZ* expression was calculated in Miller Units reported as absolute values. Predator strains WT;*SsTEF1*p-*lacZ* (G218) and WT;*SsCTS1*p*-lacZ* (G538) were assayed in three biological replicates, *Ssste12*; *SsCTS1*p*-lacZ* (G550 and G551) in six biological replicates and WT;*SsYPS3*p*-lacZ* (G556, G557 and G558) and *Ssste12*;*SsYPS3*p*-lacZ* (G560, G561 and G562) in nine biological replicates each; Mean±s.d.
(TIF)

S1 Table Strains used and generated in this study. (DOCX)

S2 Table Primers used in this study. (DOCX)

S3 Table Plasmids used and generated in this study. (DOCX)

S4 Table Quantitative predation assays. (XLSX)

S5 Table Analysis of mating behavior. (XLSX)

S6 Table Comparative global transcriptomics–RNAseq. (XLSX)

S7 Table GO enrichment analysis of upregulated genes. (XLSX)

S8 Table Motif discovery in upregulated genes. (XLSX)

S9 Table GO enrichment analysis of downregulated genes. (XLSX)

S1 Movie Time-lapse fluorescence microscopy of predation of *Saccharomyces cerevisiae* by *Saccharomycopsis schoenii*. (AVI)

S2 Movie Use of time-lapse fluorescence microscopy to determine the site of penetration peg formation. (AVI)

S3 Movie Thigmotropism of the wild type towards a prey cell. (AVI)

S4 Movie Thigmotropism of the *Sskil1* mutant towards a prey cell. (AVI)

S5 Movie Lack of predation in the *Ssste12* mutant towards *Saccharomyces cerevisiae* prey cells. (AVI)
