# Supplementary figures and images for: Re-routing MAP kinase signaling for penetration peg formation in predator yeasts
Source: PLoS Pathog. 2024 Aug 30;20(8):e1012503. doi: 10.1371/journal.ppat.1012503 (PMC11392346; doi:10.1371/journal.ppat.1012503)

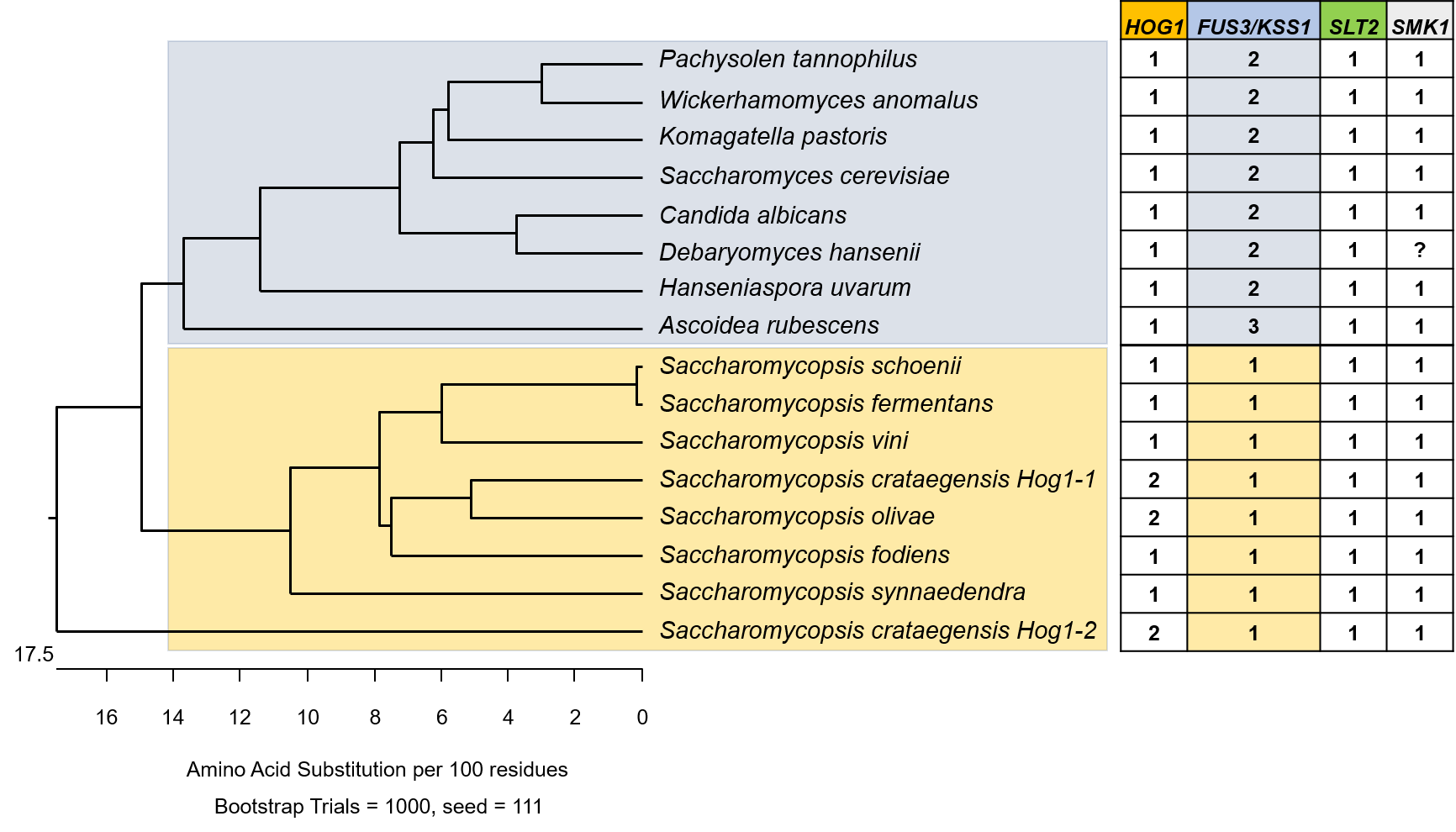

Supplement: S1 Fig — Protein tree based on Hog1 protein sequences was generated using MegAlign (DNASTAR, Madison, WI, USA) to show conservation of MAP kinase genes across diverse yeast species of Pachysolen tannophilus NRRL Y-2460 (bio project PRJNA69545), Wickerhamomyces anomalus NRRL Y-366-8, (PRJNA60493), Komagataella pastoris (PRJNA942376), Saccharomyces cerevisiae (https://www.yeastgenome.org/), Candida albicans, (http://www.candidagenome.org/), Debaryomyces hansenii CBS767 (PRJNA12410), Hanseniaspora uvarum QTX-C10 (PRJNA954297), Ascoidea rubescens DSM 1968 (PRJNA207865), Saccharomycopsis schoenii CBS 7425 (PRJNA251344), Saccharomycopsis fermentans CBS 7830 (PRJNA251344), Saccharomycopsis vini CBS 4110 (PRJNA977123), Saccharomycopsis crataegensis CBS 6448 (PRJNA977123), Saccharomycopsis olivae CBS 12701 (PRJNA736342), Saccharomycopsis fodiens CBS 8332 (PRJNA251344), Saccharomycopsis synnaedendra CBS 7763 (PRJNA977123). MAP kinases were identified using blast searches of genome sequences at NCBI (https://blast.ncbi.nlm.nih.gov/Blast.cgi). Map kinase signatures were predicted using the scan prosite tool (https://prosite.expasy.org/). Proteins that carried a MAP kinase signature (PS01351) were reciprocally blasted against S. cerevisiae using blastp at the Saccharomyces Genome Database (SGD; https://www.yeastgenome.org/). Smk1 protein orthologs were identified using ScSmk1 as query in blast searches. (TIF) [file ppat.1012503.s001.tif]

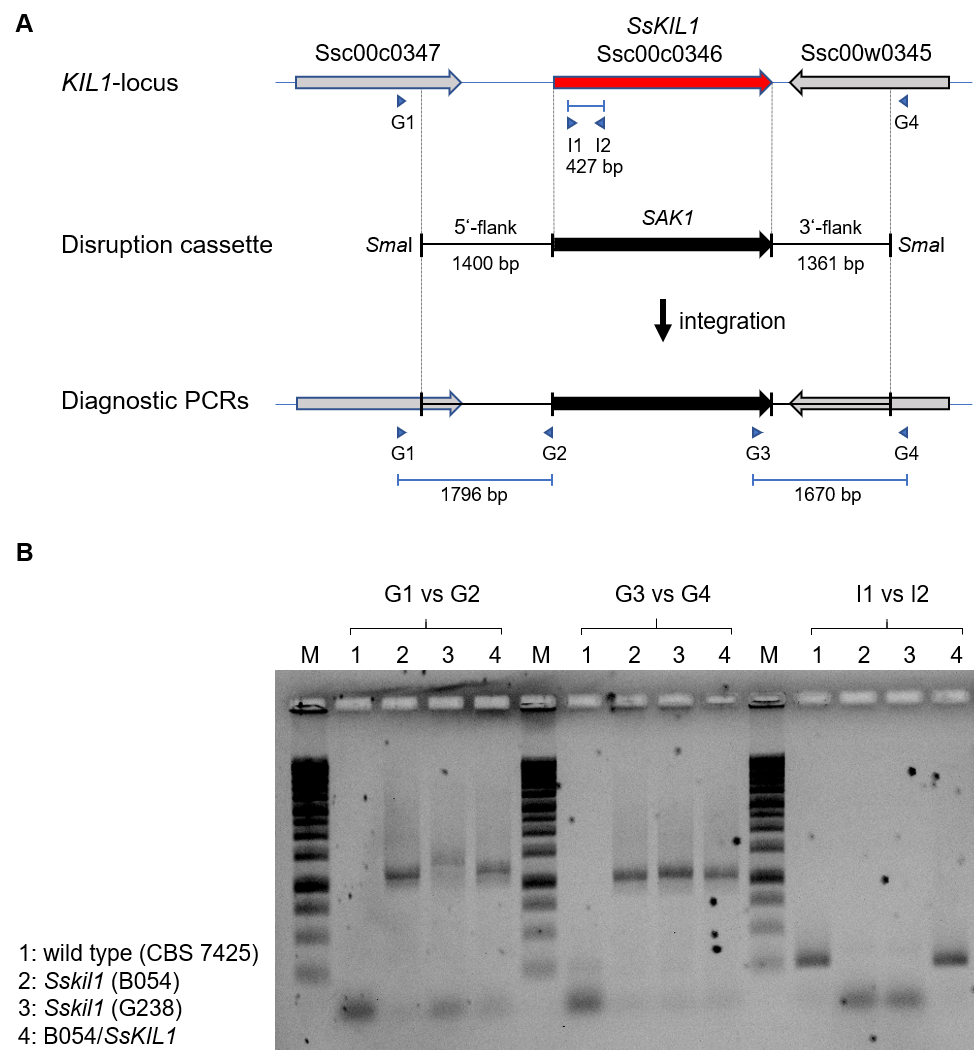

Supplement: S2 Fig — (A) Schematic representation of the SsKIL1 locus with adjacent gens, the disruption cassette containing SAK1, a dominant selectable marker gene providing resistance against [53] and the SsKIL1-locus after deletion of the SsKIL1-ORF and integration of the cassette. The 5’- and 3’-homology regions are marked as well as locus- and marker-specific primers used diagnostic PCRs to verify correct integration with the indicated expected sizes. (B) Gel image with diagnostic PCRs of the Sskil1 mutants and the complemented strain compared to wild type. Correct transformants showed G1-G2 and G3-G4 bands. Sskil1 mutants lacked the I1-I2 SsKIL1-internal band, which is present in the wild type and the complemented strain. (TIF) [file ppat.1012503.s002.tif]

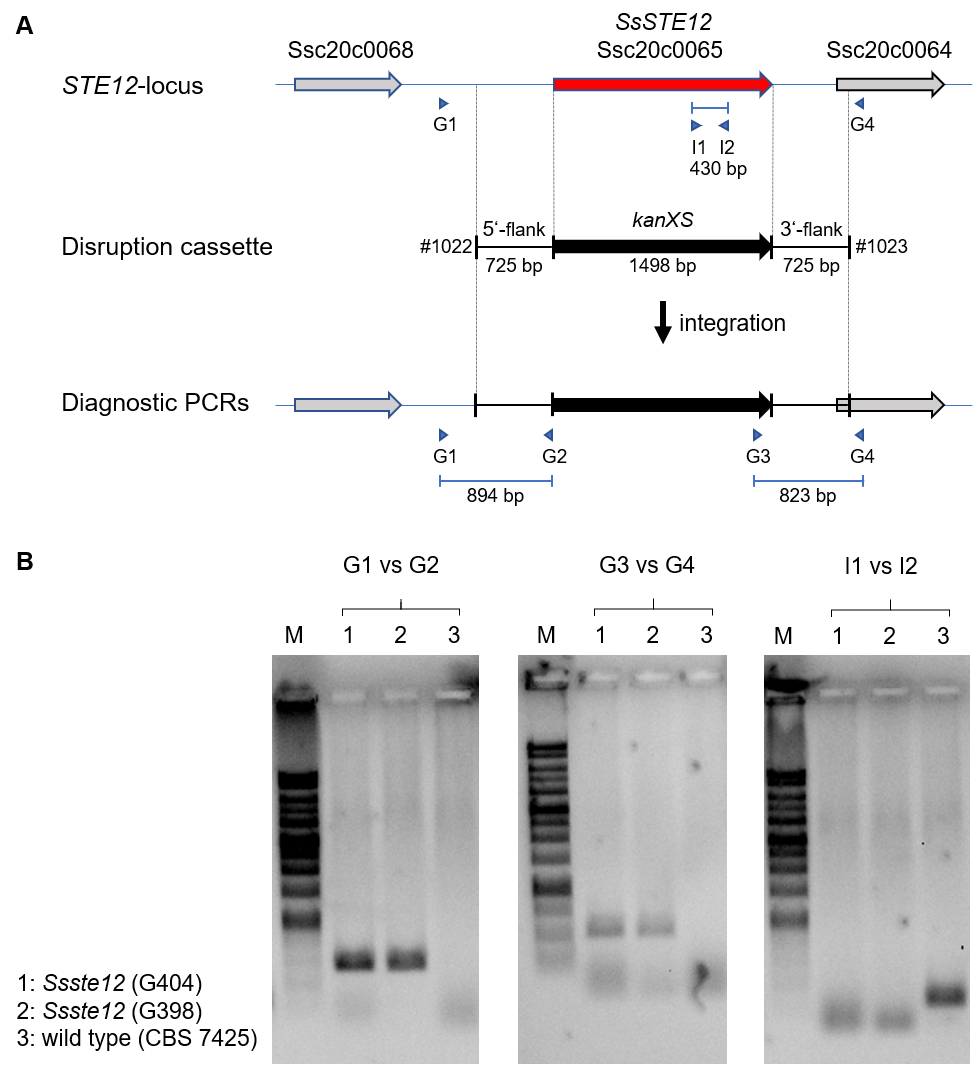

Supplement: S3 Fig — (A) Schematic representation of the SsSTE12 locus with adjacent gens, the disruption cassette containing kanXS, a synthetic dominant selectable marker gene consisting of the SsPGK1 promoter and the kanamycin resistance ORF derived from YES1 [53] and the SsKIL1-locus after deletion of the SsSTE12-ORF and integration of the cassette. The 5’- and 3’-homology regions are marked as well as locus- and marker-specific primers used diagnostic PCRs to verify correct integration with the indicated expected sizes. (B) Gel image with diagnostic PCRs of the Ssste12 mutants and the complemented strain compared to wild type. Correct transformants showed G1-G2 and G3-G4 bands and lacked an I1-I2 SsSTE12-internal band, which is present in the wild type. (TIF) [file ppat.1012503.s003.tif]

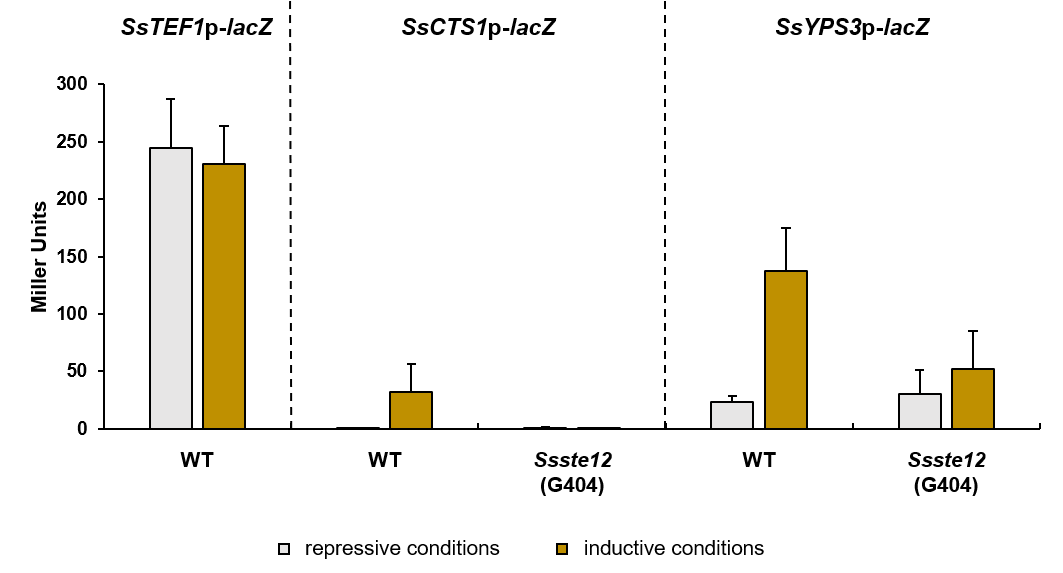

Supplement: S4 Fig — Promoters of the predation response genes Ssc08w0210-YPS3p and Ssc02c0119-CTS1p were tested for conditional regulation under predation conditions using a liquid-phase β-galactosidase assay. Saccharomycopsis strains were trialed on YPD without prey cells (repressive conditions) and on SD with prey cells (inductive conditions) as described in Materials and methods. The lacZ expression was calculated in Miller Units reported as absolute values. Predator strains WT;SsTEF1p-lacZ (G218) and WT;SsCTS1p-lacZ (G538) were assayed in three biological replicates, Ssste12; SsCTS1p-lacZ (G550 and G551) in six biological replicates and WT;SsYPS3p-lacZ (G556, G557 and G558) and Ssste12;SsYPS3p-lacZ (G560, G561 and G562) in nine biological replicates each; Mean±s.d. (TIF) [file ppat.1012503.s004.tif]
