# Supplementary material for: Re-routing MAP kinase signaling for penetration peg formation in predator yeasts
Source: PLoS Pathog. 2024 Aug 30;20(8):e1012503. doi: 10.1371/journal.ppat.1012503 (PMC11392346; doi:10.1371/journal.ppat.1012503)
Supplement: S1 Table — (DOCX) [file ppat.1012503.s005.docx]

**S1 Table.** Strains used and generated in this study.

| **Strain** | **Genotype** | **Source** |
| --- | --- | --- |
| CBS 7425 | *Saccharomycopsis schoenii* wild type | Westerdijk Institute |
| NRRL YB-2401 | *Saccharomycopsis schoenii* wild type | ARS Culture Collection (NRRL) |
| G164 | NRRL YB-2401, *SsH4*-*GFP* | This study |
| B054 | CBS 7425, *Sskil1::SAK1* | This study |
| G238 | CBS 7425, *Sskil1::SAK1* | This study |
| B268 | *B054, Sskil1::SAK1/SsKIL1* | This study |
| G398 | CBS 7425, *Ssste12::kanXS* | This study |
| G404 | CBS 7425, *Ssste12::kanXS* | This study |
| G193 | CBS 7425, *YES2* | This study |
| G498 | CBS 7425, *YES3* | This study |
| G470 | B054, *YES2* | This study |
| G473 | B054, *YES3* | This study |
| G463 | G404, *YES2* | This study |
| G465 | G404, *YES3* | This study |
| G218 | CBS 7425, *SsTEF1*p-*lacZ*, *YES1* | [1] |
| G538 | CBS 7425, *SsCTS1*p-*lacZ-YES2* | This study |
| G550 | G404, *SsCTS1*p-*lacZ-YES2* | This study |
| G551 | G404, *SsCTS1*p-*lacZ-YES2* | This study |
| G556 | CBS 7425, *SsYPS3*p-*lacZ-YES2* | This study |
| G557 | CBS 7425, *SsYPS3*p-*lacZ-YES2* | This study |
| G558 | CBS 7425, *SsYPS3*p-*lacZ-YES2* | This study |
| G560 | G404, *SsYPS3*p-*lacZ-YES2* | This study |
| G561 | G404, *SsYPS3*p-*lacZ-YES2* | This study |
| G562 | G404, *SsYPS3*p-*lacZ-YES2* | This study |
| BY4741 | *Saccharomyces cerevisiae* S288C  *his3Δ1; leu2Δ0; met15Δ0; ura3Δ0* | Euroscarf |
| CEN.PK | *Saccharomyces cerevisiae*  CEN.PK113-7d x CEN.PK113-1A | Euroscarf |
| DHY6 | *Saccharomyces cerevisiae H4-GFP* | Lab collection |

Reference

1. Rij M, Wendland J. Use of the *Saccharomycopsis schoenii MET17* promoter for regulated heterologous gene expression. Curr Genet. 2024; 70(1):9. PMID:38951203.
