# Supplementary material for: Re-routing MAP kinase signaling for penetration peg formation in predator yeasts
Source: PLoS Pathog. 2024 Aug 30;20(8):e1012503. doi: 10.1371/journal.ppat.1012503 (PMC11392346; doi:10.1371/journal.ppat.1012503)
Supplement: S2 Table — (DOCX) [file ppat.1012503.s006.docx]

**S2 Table**. Primers used in this study.

| **Primer name** | **Sequence (5‘-3‘)** |
| --- | --- |
| 18-I1-*SAK1* | ATGGGTAAGGAAAAGACTCACG |
| 44-5‘-*SsKIL1*-A | GCCATTGAACTCGCAGCTACC |
| 45-5'-*SsKIL1*-B | CGGTAGAAGTTTAAGCTGAAC |
| 46-3'-*SsKIL1*-A | CAAAGAAGTGATGGCTCCTC |
| 47-3'-*SsKIL1*-B | CCGATTGGTGGTAACCAGTTG |
| 48-5'-*SsKIL1*-A-415-adaptor | **TGTAAAACGACGGCCAGTGAGCGCGCGTAATACGACTCACTATAG**CCCGGGCCATTGAACTCGCAGCTACC |
| 49-5'-*SsKIL1*-B-*SAK1*-adaptor | **GACCTGCAGCGTACGAAGCTTCAGGCATGACACGAGTGATGTCAT**CGGTAGAAGTTTAAGCTGAAC |
| 50-3'-*SsKIL1*-A-*SAK1*-adaptor | **CTCGAATTCATCGATGATATCAGAATTCTTTCTGTATGTAAGTCT**CAAAGAAGTGATGGCTCCTC |
| 51-3'-*SsKIL1*-B-415-adaptor | **GGAAACAGCTATGACCATGATTACGCCAAGCGCGCAATTAACCCT**CCCGGGCCGATTGGTGGTAACCAGTTG |
| 80-G1-*SsKIL1* | GGTGGTGAACAACCATACG |
| 81-G4-*SsKIL1* | GTGACCCACTGTTCAACCATG |
| 82-I1-*SsKIL1* | GTGCTTATGGTATCGTGTGC |
| 83-I2-*Ss*-*KIL1* | GAAGCTATGGAACGAGCGAG |
| 96-S1-*SAK1*-adaptor | **ATGACATCACTCGTGTCATGCC**TGAAGCTTCGTACGCTGCAGGTC |
| 97-S2-*SAK1*-adaptor | **AGACTTACATACAGAAAGAAT**TCTGATATCATCGATGAATTCGAG |
| 107-3'-*SsTEF1*p | CTATAAAAAATGTTAGTATGGAG |
| 223-G2-*LacZ* | ATGATCTGAGTGAGCATCAACAG |
| 251-G3-*SsTEF1*p | CTGCTCACACCGTAAATCATTAG |
| 445-F-*SsKIL1*+adaptor | **TGTAAAACGACGGCCAGTGAGCGCGCGTAATACGACTCACTATAG**CTCGAGGTACTATCACTACCGCATG |
| 447-R-*SsKIL1*+adaptor | **TTCAAGCTGTAAAAATTCCGGTTGATTTGATGTTATTCTGGACGG**GCAGACTTATGGTATATTCT |
| 1018-I1-*SsSTE12* | ATCCAGCACTTGCTCTCCAAC |
| 1019-I2-*SsSTE12* | CCAGCAAGGAAATCCAGTGC |
| 1020-G1-*SsSTE12* | CTACACTCAGTCGAAGCAGG |
| 1021-G4-*SsSTE12* | CACATCTCCCTGAGCTTCTG |
| 1026-G2-*SsPGK1*p | GCATAGCAAGTTCCAAACTAG |
| 1022-5'-*SsSTE12*-A | TCCTCACCGTATGGGCAGCTG |
| 1023-3'*SsSTE12*-B | ATCACTGAAGAGACGTAGTC |
| 1251-S1-Ssc08w0210-*YPS3* | **TGTAAAACGACGGCCAGTGAGCGCGCGTAATACGACTCACTATAG**GTATTAGTCTGCCTTATACTTG |
| 1252-S2-Ssc08w0210-*YPS3* | **CTTTGGATCGTTTAAATAAGTTTGAATTTTTTCAGTCATGTTCAT**TGTAGAAGAGATTTGTTGTAATTG |
| 1253-I1-Ssc08w0210-*YPS3* | GTTATCCAGCAATAACTAGTC |
| 1256-S1-Ssc02c0119-*CTS1* | **TGTAAAACGACGGCCAGTGAGCGCGCGTAATACGACTCACTATAG**CATACCTTGGAATATCTACGG |
| 1257-S2-Ssc02c0119-*CTS1* | **CTTTGGATCGTTTAAATAAGTTTGAATTTTTTCAGTCATGTTCAT**GGTTCAAATTAACAATGATTGTAAC |
| 1258-I1-Ssc02c0119-*CTS1* | GCCGCATATCCACGTGCGTG |
| 1272-S1-Hyg marker insertion | **GCTTCATGATTGAACCACTAAATTAGGATCCGGCGCGCCAGATCT**GTCCAGAATAACATCAAATC |
| 1273-S2-Hyg marker insertion | **TTACGCCAAGCGCGCAATTAACCCTCACTAAAGGGAACAAAAGCTG**GAGCTCGACCACCTTTGATTGTAAATAG |
| 7269-G2-*SAK1* | CACATACCGTTAGATTCGTAG |
| 7270-G3-*SAK1* | GCAATTCCACTTGATGCTTGATG |
| 7272-I2-*SAK1* | CAGGAATAGAGTGCAAACGACG |
| **Bold:** adaptor sequences for *in vivo* cloning, underlined: *Sma*I restriction site, underlined: *Sac*I restriction site | |
