# Supplementary material for: Re-routing MAP kinase signaling for penetration peg formation in predator yeasts
Source: PLoS Pathog. 2024 Aug 30;20(8):e1012503. doi: 10.1371/journal.ppat.1012503 (PMC11392346; doi:10.1371/journal.ppat.1012503)
Supplement: S3 Table — (DOCX) [file ppat.1012503.s007.docx]

**S3 Table.** Plasmids used and generated in this study.

| **Plasmid** | **Features** | **Source** |
| --- | --- | --- |
| pGEM-T | pGEM-T Easy Vector System | Promega |
| pRS415 | *amp*, *LEU2* | Lab collection |
| pUC57-*SAK1* | *amp*, *SAK1* | Genscript |
| E020-pGEM-*SsKIL1-*5‘-flank | *amp* | This study |
| E021-pGEM-*SsKIL1*-3‘-flank | *amp* | This study |
| E034-pRS415-*SsKIL1* disruption | *amp*, *LEU2*, *SAK1* | This study |
| E068-pRS415-*YES2* | *amp*, *LEU2*, *YES2* | [1] |
| E070-pRS415-*YES3* | *amp*, *LEU2*, *YES3* | [1] |
| E074-pRS417-*SsMET17*p-*lacZ*-*SAK1* | *amp, lacZ, SAK1* | [1] |
| E177-pGEM-*SsKIL1*p | *amp* | This study |
| E178-pGEM-*SsKIL1*-ORF | *amp* | This study |
| E182-pRS415-*SsKIL1* complementation | *amp*, *LEU2*, *YES2* | This study |
| E196-pUC57-*SsH4*-GFP | *amp*, *YES1* | Genscript |
| E265-pGADT7-*SsSTE12* disruption | *amp*, *LEU2*, *kanXS** | BioCat |
| E324-pRS417-*SsCTS1*p-*lacZ*-*YES2* | *amp, lacZ, YES2* | This study |
| E326-pRS417-*SsYPS3*p-*lacZ*-*YES2* | *amp, lacZ, YES2* | This study |
| * *kanXS* is composed of *SsPGK1*p and *kan*ORF derived from *YES1* [1]. | | |

Reference

1. Kayacan Y, Griffiths A, Wendland J. A script for initiating molecular biology studies with non-conventional yeasts based on *Saccharomycopsis* *schoenii*. Microbiol Res. 2019; 229: 126342. PMID:31536874.
